# Supplementary material for: Preproinsulin Designer Antigens Excluded from Endoplasmic Reticulum Suppressed Diabetes Development in NOD Mice by DNA Vaccination
Source: Mol Ther Methods Clin Dev. 2018 Dec 13;12:123–33. doi: 10.1016/j.omtm.2018.12.002 (PMC6319196; doi:10.1016/j.omtm.2018.12.002)
Supplement: Document S1. Figures S1–S5 [file mmc1.pdf]

OMTM, Volume *12*

Supplemental Information

Preproinsulin Designer Antigens Excluded from Endoplasmic Reticulum Suppressed Diabetes Development in NOD Mice by DNA Vaccination

Katja Stifter, Cornelia Schuster, Jana Krieger, Andreas Spyrantis, Bernhard Otto Boehm, and Reinhold Schirmbeck

a

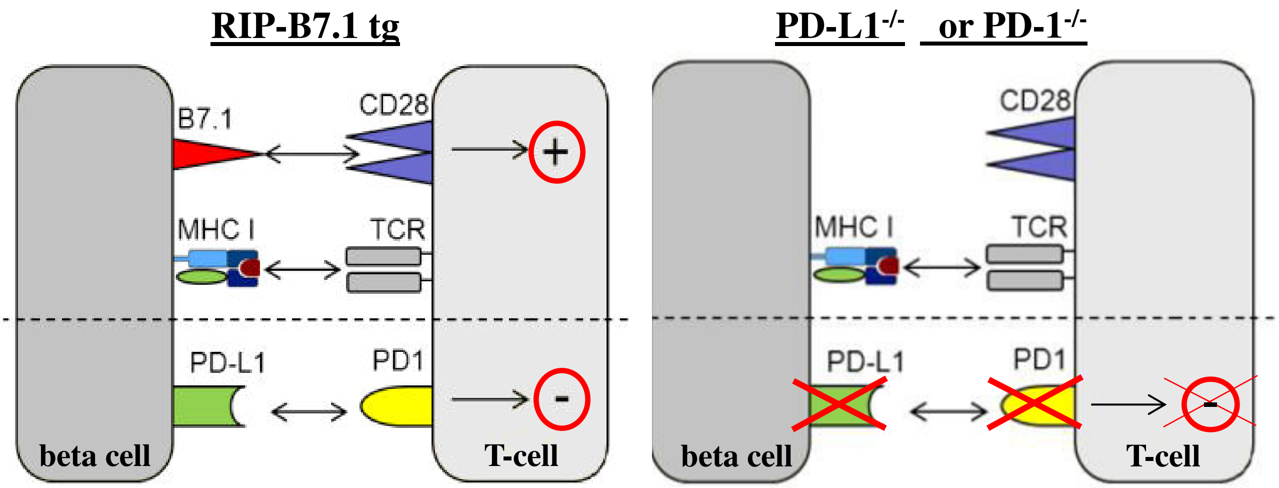

b

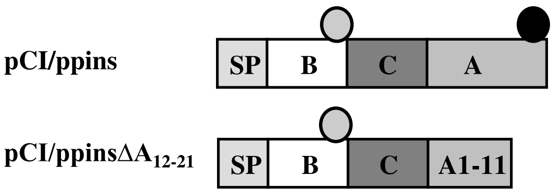

SP 1-24:ppins $_{1\text{-}24}$ **B 1-30:ppins** $_{25\text{-}54}$ ***RR*** **C 1-31:ppins** $_{57\text{-}87}$ ***KR*** **A 1-21:ppins** $_{90\text{-}110}$

● **K^b/A12-21 : SLYQLENYCN (ppins 101-110)**

○ **K^b/B22-29: RGFFYTPM (ppins 46-53)**

c

RIP-B7.1 tg	K^b/A_{12-21}	K^b/B_{22-29}	diabetes
ppins	+	-	+
ppinsΔA_{12-21}	-	+	+

PD-1/PD-L1$^{-/-}$	K^b/A_{12-21}	K^b/B_{22-29}	diabetes
ppins	+	-	+
ppinsΔA_{12-21}	-	-	-

Supplementary Fig. S1

Diabetes induction in the RIP-B7.1 tg mouse model. (a) Schematic presentation of the beta cell and T cell interaction in RIP-B7.1 tg, PD-L1$^{-/-}$ and PD-1$^{-/-}$ mice. (b) Map of the pCI-encoded ppins- and ppinsΔA_{12-21}-antigens. The positions of the insulin signal peptide (SP), B-, C- and A-chain, as well as the position and sequences of the two K^b-restricted epitopes (K^b/A_{12-21} and K^b/B_{22-29}) are indicated. (c) Diabetes induction in RIP-B7.1 tg mice after pCI/ppins immunization is driven by K^b/A_{12-21}-monospecific CD8$^+$ T cells, while pCI/ppinsΔA_{12-21}-injection exclusively leads to induction of K^b/B_{22-29}-specific CD8$^+$ T cells and diabetes. In PD-L1$^{-/-}$ and PD-1$^{-/-}$ mice only pCI/ppins-immunization induces a K^b/A_{12-21}-specific diabetogenic CD8$^+$ T cell response, while pCI/ppinsΔA_{12-21}-injection induces neither K^b/B_{22-29}-specific CD8$^+$ T cells nor diabetes.

PD-L1$^{-/-}$ mice immunized with:

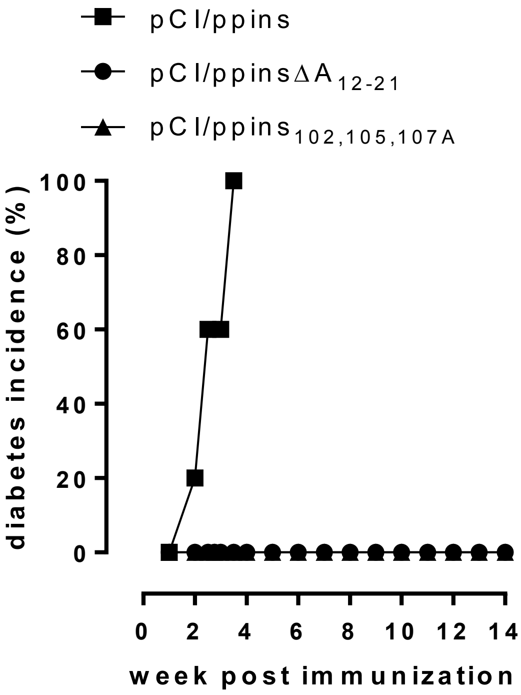

Supplementary Fig. S2
Diabetes suppressive potential of pCI/ppinsΔA_{12-21} and pCI/$ppins_{102,105,107A}$ in PD-L1$^{-/-}$ mice. PD-L1$^{-/-}$ mice were immunized with pCI/ppins, pCI/ppinsΔA_{12-21} or pCI/$ppins_{102,105,107A}$ (n=5 per group) and diabetes development was followed by regular blood glucose measurements and determination of the diabetes incidence (%).

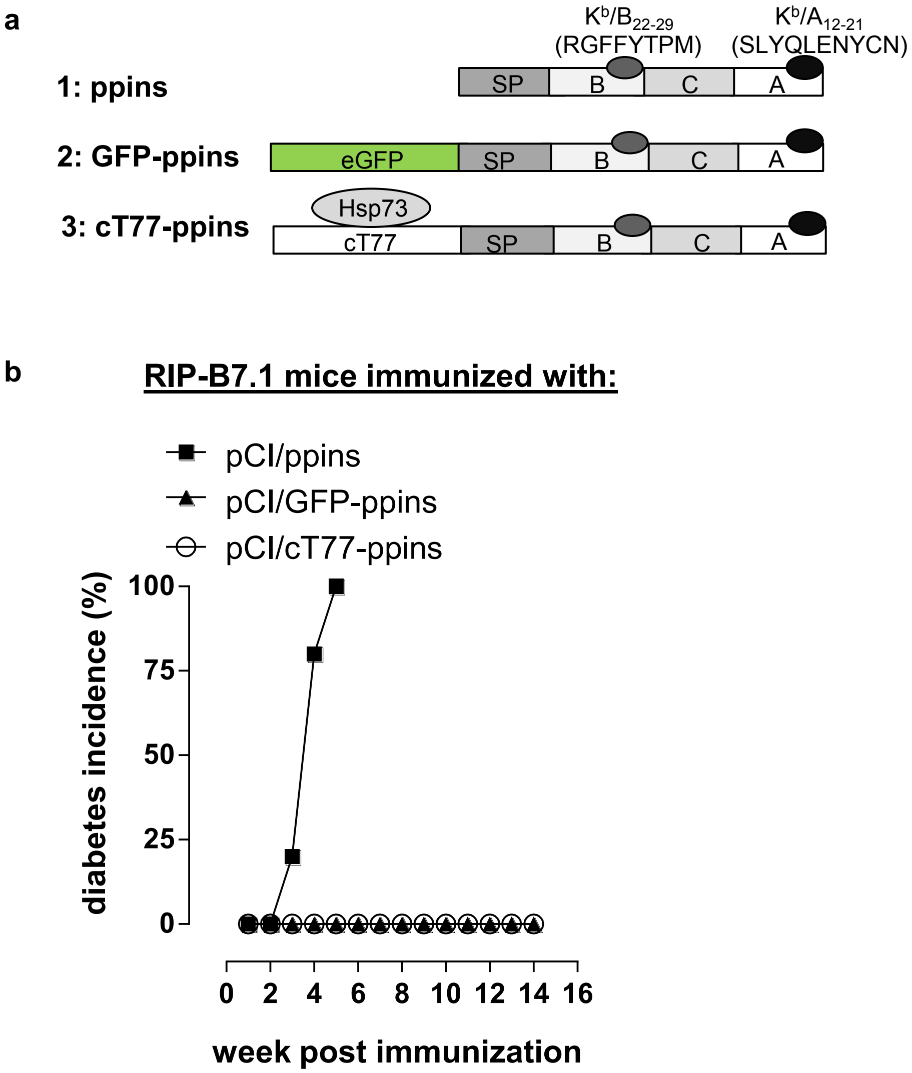

Supplementary Fig. S3

Evaluation of the diabetogenic potential of ppins-designer antigens GFP-ppins and cT77-ppins in RIP-B7.1 tg mice. (a) Maps of ppins and the designer antigens GFP-ppins and cT77-ppins. The positions and aa sequences of the ppins epitopes K^b/A_{12-21} and K^b/B_{22-29} are shown. The binding of Hsp73 to the cT77-domain (77 aa fragment of the SV40 large T-antigen) is indicated. (b) Diabetes development after pCI/GFP-ppins- or pCI/cT77-ppins-immunization of RIP-B7.1 tg mice, compared to pCI/ppins-immunization (n=5 per group), was followed by regular determination of the blood glucose levels and cumulative diabetes incidences (%).

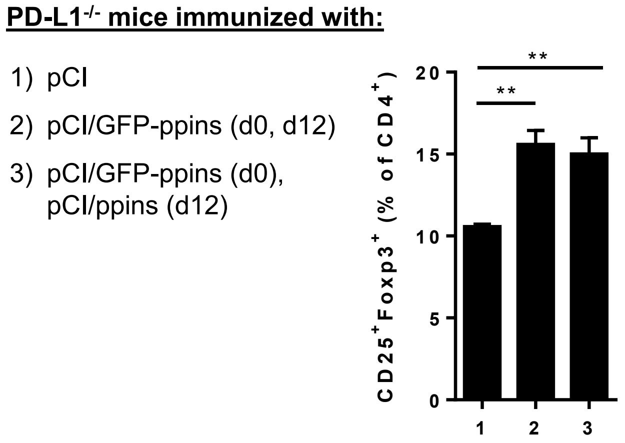

Supplementary Fig. S4

Induction of Foxp3$^+$CD25$^+$CD4$^+$ Treg cells in PD-L1$^{-/-}$ mice by pCI/GFP-ppins-vaccination. PD-L1$^{-/-}$ mice were sham-immunized (pCI, group 1, n=5), vaccinated twice (on day 0 and day 12) with pCI/GFP-ppins (group 2, n=4), or vaccinated with pCI/GFP-ppins followed by pCI/ppins-priming at day 12 post vaccination (group 3, n=4). On day 14 post pCI/ppins-injection, lymphocytes derived from pancreatic lymph nodes were stained for CD25$^+$Foxp3$^+$ regulatory CD4$^+$ T cells. Bar graphs show the frequencies of CD25$^+$Foxp3$^+$ cells among the CD4$^+$ T cell population + SD. Indicated statistically significant differences between groups 1 and 2 and between groups 1 and 3 were determined using the unpaired student's t-test. A value of (*) $p < 0.05$ was considered significant (** significant at $p < 0.01$).

NOD mice immunized with:

a) pCI/GFP-ppins

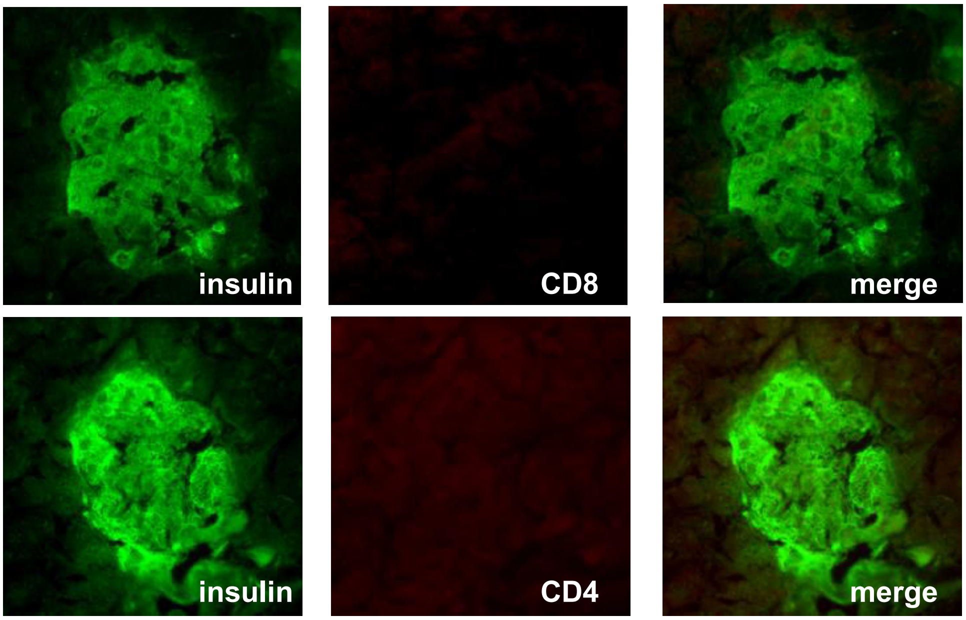

b) pCI

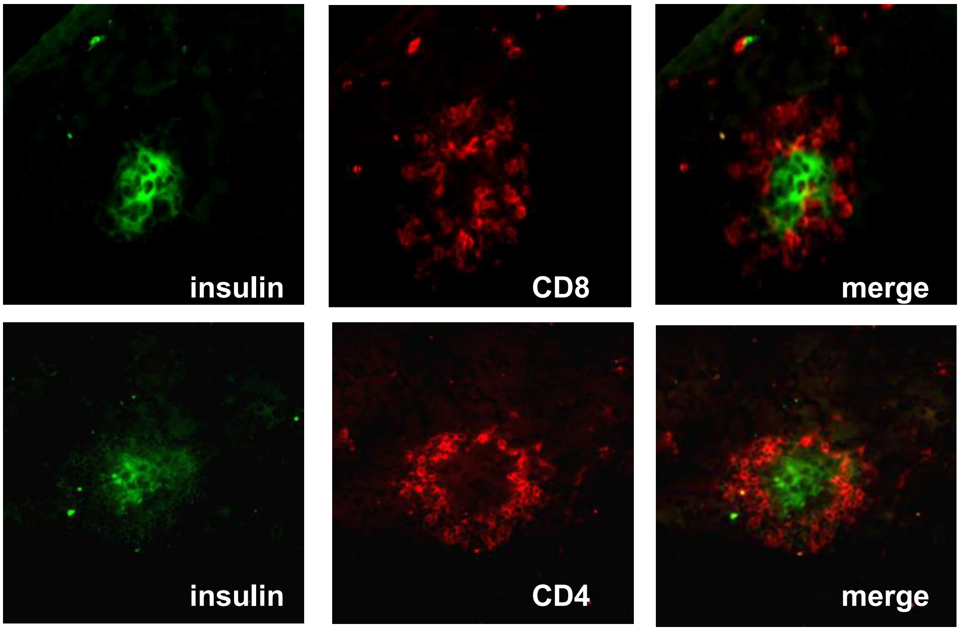

Supplementary Fig. S5

Islet-pathology of pCI/GFP-ppins- and pCI-sham-immunized NOD mice. The islet-pathology and immune cell infiltrations into pancreata of 20 weeks old healthy, pCI/GFP-ppins-immune (a) and control (injected with empty pCI vector) hyperglycemic NOD mice (b) were assessed by immunofluorescence staining with mAbs detecting insulin, $CD8^+$ T cells or $CD4^+$ T cells.
