# Supplementary material for: Preproinsulin Designer Antigens Excluded from Endoplasmic Reticulum Suppressed Diabetes Development in NOD Mice by DNA Vaccination
Source: Mol Ther Methods Clin Dev. 2018 Dec 13;12:123–33. doi: 10.1016/j.omtm.2018.12.002 (PMC6319196; doi:10.1016/j.omtm.2018.12.002)
Supplement: Document S2. Article plus Supplemental Information [file mmc2.pdf]

Molecular Therapy
Methods & Clinical Development
Original Article

Preproinsulin Designer Antigens Excluded from Endoplasmic Reticulum Suppressed Diabetes Development in NOD Mice by DNA Vaccination

Katja Stifter,[1] Cornelia Schuster,[1,2] Jana Krieger,[1] Andreas Spyrantis,[1] Bernhard Otto Boehm,[3,4] and Reinhold Schirmbeck[1]

[1]Department of Internal Medicine I, Ulm University Hospital, Albert Einstein Allee 23, 89081 Ulm, Germany; [2]Department of Dermatology, University Medical Center of the Johannes Gutenberg-University, Mainz, Germany; [3]Lee Kong Chian School of Medicine, Nanyang Technological University, Singapore, Singapore; [4]Imperial College London, London, UK

DNA vaccines against autoimmune type 1 diabetes (T1D) contain a nonpredictable risk to induce autoreactive T cell responses rather than a protective immunity. Little is known if (and how) antigen expression and processing requirements favor the induction of autoreactive or protective immune responses by DNA immunization. Here, we analyzed whether structural properties of preproinsulin (ppins) variants and/or subcellular targeting of ppins designer antigens influence the priming of effector CD8$^+$ T cell responses by DNA immunization. Primarily, we used H-2^b RIP-B7.1 tg mice, expressing the co-stimulator molecule B7.1 in beta cells, to identify antigens that induce or fail to induce autoreactive ppins-specific (K^b/A$_{12\text{-}21}$ and/or K^b/B$_{22\text{-}29}$) CD8$^+$ T cell responses. Female NOD mice, expressing the diabetes-susceptible H-2^{g7} haplotype, were used to test ppins variants for their potential to suppress spontaneous diabetes development. We showed that ppins antigens excluded from expression in the endoplasmic reticulum (ER) did not induce CD8$^+$ T cells or autoimmune diabetes in RIP-B7.1 tg mice, but efficiently suppressed spontaneous diabetes development in NOD mice as well as ppins-induced CD8$^+$ T cell-mediated autoimmune diabetes in *PD-L1*$^{-/-}$ mice. The induction of a ppins-specific therapeutic immunity in mice has practical implications for the design of immune therapies against T1D in individuals expressing different major histocompatibility complex (MHC) I and II molecules.

INTRODUCTION

DNA vaccination is a potent strategy to induce autoreactive effector CD8$^+$ T cell responses against self-antigens, e.g., preproinsulin (ppins) in pancreatic beta cells, but can also lead to prophylactic immune responses calibrating T cell-mediated autoreactivity.[1,2] In both scenarios, *de novo* priming of immune responses against the major beta cell autoantigen ppins is mandatory. However, little is known about the antigen expression and processing requirements that favor either the induction of autoreactive or protective immune responses. RIP-B7.1 tg mice expressing the proinflammatory immune checkpoint molecule B7.1 (CD80)[3] have been useful to study *de novo* priming of antigen-specific CD8$^+$ T cells by DNA immunization and their subsequent pathogenic crosstalk with islet beta cells.[4–10] Transgenic expression of the B7.1 molecule in beta cells of RIP-B7.1 tg mice converts these cells into "professional-like" antigen-presenting cells (APCs) (Figure S1A). As a consequence, B7.1$^+$ beta cells could directly interact with CD28 on T cells and stimulate *de novo*-primed or adoptively transferred autoreactive CD8$^+$ T cells, finally leading to a CD8$^+$ T cell-mediated destruction of beta cells and development of clinically overt disease, mimicking largely the situation in humans afflicted with T1D.[7,8] Priming of ppins-specific autoreactive CD8$^+$ T cells in RIP-B7.1 tg mice by DNA immunization did not require CD4$^+$ T cell help.[7,8] Therefore, RIP-B7.1 tg mice provide an attractive model system to identify beta-cell-specific antigens that are targeted by autoreactive T cells[4,7–9,11] and to identify designer antigens with the potential to mitigate autoreactive CD8$^+$ T cells.[6]

A single injection of ppins DNA selectively induced K^b/A$_{12\text{-}21}$-monospecific CD8$^+$ T cells, whereas a mutant ppinsΔA$_{12\text{-}21}$ antigen (lacking the K^b/A$_{12\text{-}21}$ epitope) induced insulin B-chain K^b/B$_{22\text{-}29}$-monospecific CD8$^+$ T cells in RIP-B7.1 tg mice (Figures S1B and S1C).[8] A simple manipulation of ppins thus generated an altered repertoire of autoreactive CD8$^+$ T cells. On the other hand, ppins designer antigens targeted to the cytosol and/or the nucleus and thus excluded from the endoplasmic reticulum (ER) did not induce autoimmune diabetes in RIP-B7.1 tg mice.[6] Expression of ppins designer antigens in the ER was a prerequisite to induce both K^b/A$_{12\text{-}21}$- and K^b/B$_{22\text{-}29}$-specific effector CD8$^+$ T cells in RIP-B7.1. tg mice by DNA immunization. Expression and processing of proteins in the ER and the secretory route might increase the presentation efficacy of autoantigen-derived epitopes that bind major histocompatibility complex (MHC) class I molecules with low affinity,[12–16] because they must not compete with the bulk of antigenic peptides generated in the conventional MHC I antigen-presentation pathway for transporters associated

Received 8 October 2018; accepted 3 December 2018;
https://doi.org/10.1016/j.omtm.2018.12.002.

Correspondence: Reinhold Schirmbeck, Department of Internal Medicine I, Ulm University Hospital, Albert Einstein Allee 23, 89081 Ulm, Germany.
E-mail: reinhold.schirmbeck@uni-ulm.de

This is an open access article under the CC BY license (http://creativecommons.org/licenses/by/4.0/).

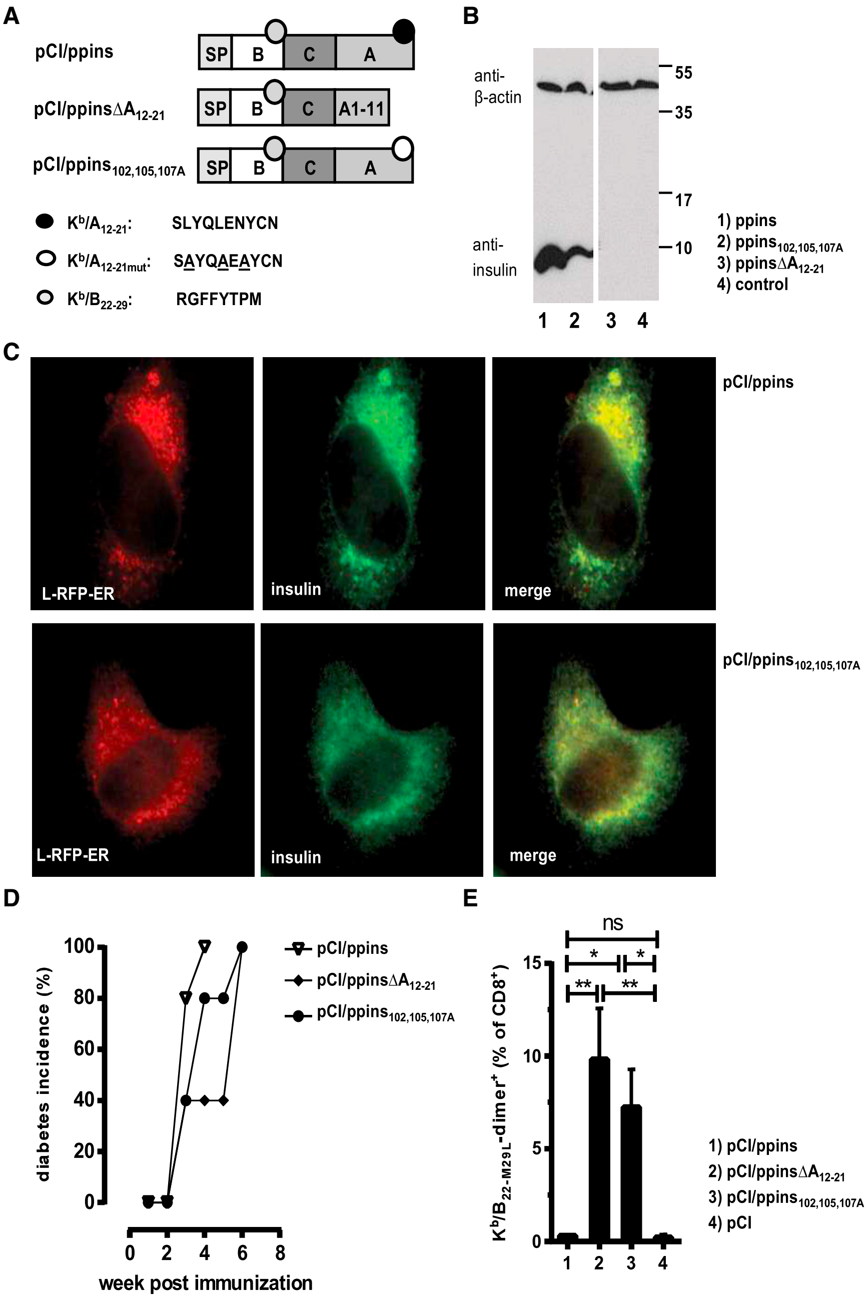

Figure 1. Diabetes Induction in RIP-B7.1 tg Mice by Ppins-Derived Antigens Lacking the Dominant K^b/A_{12-21} Epitope

(A) Schematic presentation of the K^b/A_{12-21} deleted or mutated antigens ppinsΔA_{12-21} and ppins$_{102,105,107A}$.

(B) HEK293 cells were transiently transfected with pCI/ppins (lane 1), pCI/ppins$_{102,105,107A}$ (lane 2), pCI/ppinsΔA_{12-21} (lane 3), or control pCI DNA (lane 4). At 48 hr post-transfection, cells were lysed and total cell extracts were subjected to SDS-PAGE (12.5%) followed by anti-insulin- and anti-beta-actin-specific western blotting.

(C) Alternatively, HeLa cells were co-transfected with pCI/ppins or pCI/ppins$_{102,105,107A}$ DNA and an ER-targeted, red fluorescent protein (L-RFP-ER)-expressing vector. Ppins or insulin staining was performed using anti-insulin and FITC-conjugated anti-rabbit IgG.

(D) RIP-B7.1 tg mice (n = 5 per group) were immunized with pCI/ppins (group 1), pCI/ppinsΔA_{12-21} (group 2), or pCI/ppins$_{102,105,107A}$ (group 3), and diabetes development was followed by determination of the blood glucose levels. Cumulative diabetes incidences (%) are shown.

(E) Frequencies of K^b/B_{22-29}-specific $CD8^+$ T cells in the pancreata of diabetic, pCI/ppins (group 1, n = 7)-, pCI/ppinsΔA_{12-21} (group 2, n = 7)-, pCI/ppins$_{102,105,107A}$-immune (group 3, n = 7), and sham-immunized (pCI) (group 4; n = 7) RIP-B7.1 tg mice were determined by $K^b/B_{22\text{-}M29L}$-dimer staining. Bar graphs represent the mean percentage of $K^b/B_{22\text{-}M29L}$-dimer$^+$ cells among the $CD8^+$ T cell population ± SD. Statistical analysis was performed using a repeated-measures one-way ANOVA followed by Tukey's multiple comparisons. *$p < 0.05$; **$p < 0.01$; ns, not significant.

with antigen presentation (TAP)-dependent transport into the ER. At least the K^b/A_{12-21} epitope harbors a very low affinity for the K^b molecule.[7] The K^b/A_{12-21} epitope represents the extreme COOH terminus of the ppins molecule (i.e., the insulin A-chain; Figure 1A) and hence does not require COOH-terminal processing for loading MHC class I K^b molecules in the ER.[6] However, K^b/A_{12-21}-specific $CD8^+$ T cells were also induced by COOH terminally extended ppins fusion antigens expressed in the ER (e.g., in a pCI/ppins-GFP vector), but not when excluded from ER (e.g., in a pCI/GFP-ppins vector), indicating that the epitope position at the COOH terminus is less important for its antigenicity than antigen targeting to the ER.[6] The insulin B-chain K^b/B_{22-29} epitope is localized close to the B/C junction of ppins (Figure 1A) and therefore requires both processing at the COOH and NH_2 terminus. We thus hypothesized that intrinsic properties of the antigen and/or antigen processing and presentation in the ER contribute to the induction of autoreactive ppins-specific $CD8^+$ T cell responses.[6,17]

Interestingly, the pCI/ppins vector selectively primed K^b/A_{12-21}-specific effector $CD8^+$ T cells in C57BL/6J (B6) mice, but they did not induce autoimmune diabetes.[7,8] However, K^b/A_{12-21}-specific $CD8^+$ T cells primed in B6 mice induced autoimmune diabetes after adoptive transfer into RIP-B7.1 tg hosts or directly after treatment of pCI/ppins-immune B6 mice with anti PD-L1 antibody.[7] In contrast, the pCI/ppinsΔA_{12-21} vector did not induce K^b/B_{22-29}-specific $CD8^+$ T cells in B6 mice.[7,8] Furthermore, pCI/ppins but not pCI/ppinsΔA_{12-21} primed autoreactive $CD8^+$ T cells and autoimmune diabetes in co-inhibition-deficient *PD-1*$^{-/-}$ or *PD-L1*$^{-/-}$ mice.[7,8] This suggested that priming and/or expansion of K^b/B_{22-29}- but not K^b/A_{12-21}-specific $CD8^+$ T cells critically depended on B7.1-mediated co-stimulatory

signals from tg beta cells (Figures S1B and S1C).[8,18] However, pCI/ppinsΔA_{12-21} injection into *PD-1*$^{-/-}$ or *PD-L1*$^{-/-}$ mice elicited a systemic Foxp3$^+$CD25$^+$CD4$^+$ Treg cell immunity that suppressed diabetes induction by a subsequent injection of the diabetogenic pCI/ppins vector.[18]

Nonobese diabetic (NOD) mice expressing the diabetes-susceptible H-2^{g7} haplotype (K^d, D^b; I-A^{g7}) have been exploited extensively to study diabetes development as well as to develop immunotherapies to prevent diabetes.[19] The MHC class II I-A^{g7} molecule in NOD mice, as specific human leukozyte antigen (HLA) haplotypes (DQ2; DQ8) in humans,[20] is a major determinant for developing disease but expressed in an otherwise nonsusceptible genetic background (B6 or *nonobese resistant* NOR/Lt mice) is not sufficient for diabetes development. Though the pace of insulitis and disease development differs substantially in man and NOD mice and many translating therapies from NOD mice to humans failed,[19] there are also several promising approaches. Peptide-based[21] and vector-DNA-based[22] immunotherapies have been successfully used in human trials. Vectors expressing proinsulin (pins) reduced the incidence of spontaneous diabetes development in NOD mice[23] and reduced the frequency of autoreactive CD8$^+$ T cells in patients with T1D.[22] However, genetic vaccination with ppins-expressing DNA accelerated spontaneous diabetes development in female NOD mice and diminished the natural diabetes resistance in male NOD mice.[4] This exemplifies that DNA vaccines against T1D contain a nonpredictable risk to induce autoreactive T cell responses rather than a protective immunity. We show here that ppins designer antigens expressed in or outside the ER exert a strong impact on induction of epitope-specific CD8$^+$ T cells by DNA immunization and the development of autoimmune diabetes in different mouse models of type 1 diabetes. In particular, ppins designer antigens excluded from expression in the ER efficiently suppressed spontaneous diabetes development in the NOD mouse model.

RESULTS

Deletion or Silencing of the ppins K^b/A$_{12-21}$ Epitope Restored Priming of K^b/B$_{22-29}$-Specific CD8$^+$ T Cells in RIP-B7.1 tg Mice

In RIP-B7.1 tg mice, injection of pCI/ppins DNA induced K^b/A$_{12-21}$- but not K^b/B$_{22-29}$-specific CD8$^+$ T cells, whereas a mutant ppinsΔA_{12-21} vector, lacking the COOH-terminal K^b/A$_{12-21}$ epitope, elicited K^b/B$_{22-29}$-specific CD8$^+$ T cells and autoimmune diabetes (Figures S1B and S1C).[7,8] Deletion of the A$_{12-21}$ sequence may generate a specifically folded ppinsΔA_{12-21} antigen, which is selectively processed for K^b/B$_{22-29}$-specific epitope presentation and critically depends on its instable, proteasome-mediated high turn-over expression, as detected in transiently transfected HEK293 cells.[8]

To determine whether intrinsic features of ppinsΔA_{12-21} played a crucial role for the priming of K^b/B$_{22-29}$-specific CD8$^+$ T cells, we generated a mutant ppins antigen, in which the K^b/A$_{12-21}$ (ppins$_{101-110}$) epitope was silenced by exchanging the amino acids at positions 102, 105, and 107 with alanine. This generated the pCI/ppins$_{102,105,107A}$ vector (Figure 1A). Ppins and ppins$_{102,105,107A}$, but not the ppinsΔA_{12-21} antigen was stably expressed and accumulated to pronounced steady-state levels in transiently transfected HEK293 cells (Figure 1B).[8] Both ppins$_{102,105,107A}$ and wild-type ppins proteins were expressed in the ER of transiently transfected HeLa cells (Figure 1C). Single injections of pCI/ppins$_{102,105,107A}$, pCI/ppinsΔA_{12-21}, or pCI/ppins vectors efficiently induced autoimmune diabetes in RIP-B7.1 tg mice (Figure 1D).[8] However, dimer$^+$ K^b/B$_{22-29}$-specific CD8$^+$ T cells were detectable in pCI/ppins$_{102,105,107A}$- and pCI/ppinsΔA_{12-21}-immune, but not in pCI/ppins-immune mice (Figure 1E).[8] K^b/A$_{12-21}$-specific CD8$^+$ T cells, reactive with either wild-type K^b/A$_{12-21}$ or mutant K^b/A$_{12\text{-}N21A}$ peptides[6] were not detectable in pCI/ppinsΔA_{12-21}[8] and pCI/ppins$_{102,105,107A}$-immune mice (data not shown). Silencing of the K^b/A$_{12-21}$ epitope in the pCI/ppins$_{102,105,107A}$ construct was further confirmed in co-inhibition-deficient *PD-L1*$^{-/-}$ mice. In these mice, only ppins constructs that trigger K^b/A$_{12-21}$-specific effector CD8$^+$ T cells induce autoimmune diabetes[7,8] and injection of pCI/ppins$_{102,105,107A}$ did not induce autoimmune diabetes (Figure S2). Overall, these findings indicated that antigen-specific properties of endogenously expressed ppinsΔA_{12-21} were not the primary cause for the priming of K^b/B$_{22-29}$-specific CD8$^+$ T cells. Minor changes in the ppins$_{102,105,107A}$ antigen thus affected *de novo* priming of autoreactive CD8$^+$ T cells in an epitope-specific manner. However, we could not exclude that the presence or absence of the K^b/A$_{12-21}$-epitope (and K^b/A$_{12-21}$-specific CD8$^+$ T cells) may also affect the priming of K^b/B$_{22-29}$-specific CD8$^+$ T cells in RIP-B7.1 tg mice, for example, by intrinsic local immune dominance phenomena.[6]

Ppins or Pins Designer Antigens Excluded from Expression in the ER Did Not Induce Autoimmune Diabetes

A central aim of our studies was to design ppins or pins antigens that do not induce autoreactive K^b/A$_{12-21}$ and K^b/B$_{22-29}$-specific CD8$^+$ T cells in RIP-B7.1 tg mice. We previously showed that ppins antigens, targeted to the cytosol and/or the nucleus by fusing the ppins sequence COOH terminally to the GFP (pCI/GFP-ppins) or to a 77-residue stress protein (Hsp73)-binding domain of the SV40 large T antigen[24] (pCI/cT77-ppins), were excluded from expression and processing in the ER and did not induce autoimmune diabetes in RIP-B7.1 tg mice (Figure S3).[6] Because these fusion antigens contain large heterologous protein sequences (Figure S3) that could induce ill-defined immune responses and thereby influence priming of autoreactive CD8$^+$ T cells, we generated a small nuclear pins construct by exchanging the sequence of the ER-targeting signal peptide of ppins (SP) with a 19-amino-acids-long SV40 T-Ag-derived nuclear localization sequence (NLS; pCI/NLS-pins) (Figure 2A). To facilitate detection of this antigen, we fused an HA-tag sequence COOH terminally in frame to the NLS-pins sequence (Figure 2A). The NLS-pins antigen was stably expressed in the nucleus of transiently transfected HEK293 cells (Figures 2B and 2C). Injection of the pCI/NLS-pins vector into RIP-B7.1 tg mice did not induce autoimmune diabetes (Figure 2D). Furthermore, no signs of CD8$^+$ T cell infiltration into islets or destruction of islets were detectable in pCI/NLS-pins-immune RIP-B7.1 tg mice by histology (Figure 2E). Overall, these findings confirmed that prevention of antigen expression in the ER did not

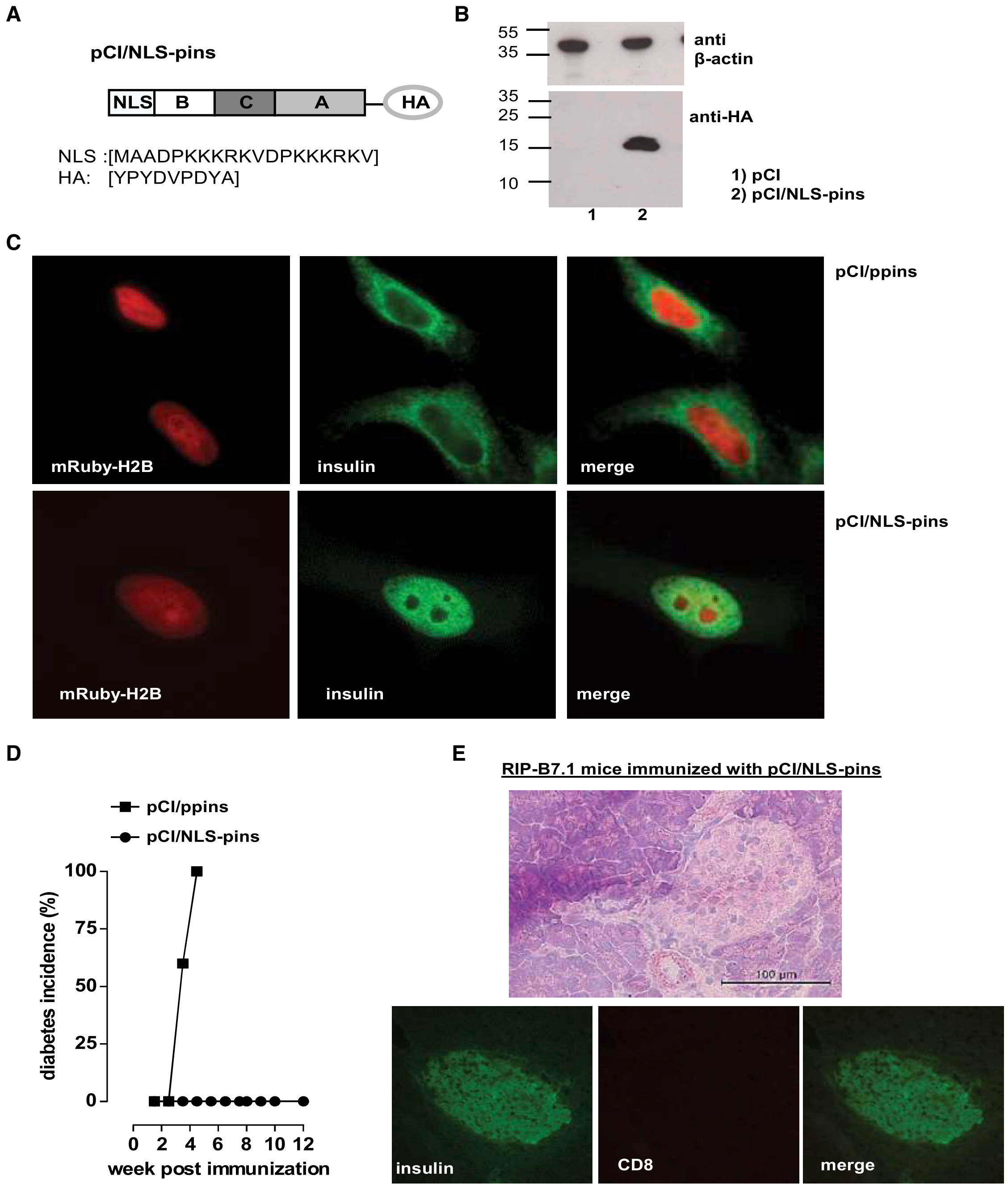

Figure 2. Characterization of the pCI/NLS-pins Vaccine

(A) Schematic presentation of the HA-tagged NLS-pins antigen. The aa sequences of the nuclear localization signal (NLS) and the HA-tag (HA) are shown in brackets.

(B) Anti-HA- and anti-beta-actin-specific western blotting was performed on total cell extracts of HEK293 cells at 48 hr post-transfection with pCI (lane 1) or pCI/NLS-pins (lane 2).

(C) HeLa cells were either co-transfected with pCI/ppins and mRuby-H2B vector or with pCI/NLS-pins and mRuby-H2B vector. Antigen (pins)-specific expression was detected using anti-insulin (H-86) and FITC-conjugated anti-rabbit IgG.

(D) Diabetes development in pCI/ppins or pCI/NLS-pins immunized RIP-B7.1 tg mice (n = 5 per group) was followed by regular determination of the blood glucose levels. Cumulative diabetes incidences (%) are shown.

(E) Pancreatic sections from healthy, pCI/NLS-pins-immune RIP-B7.1 tg mice were analyzed by H&E staining and immunofluorescence staining for insulin and $CD8^+$ T cells. μScale bar, 100 μm.

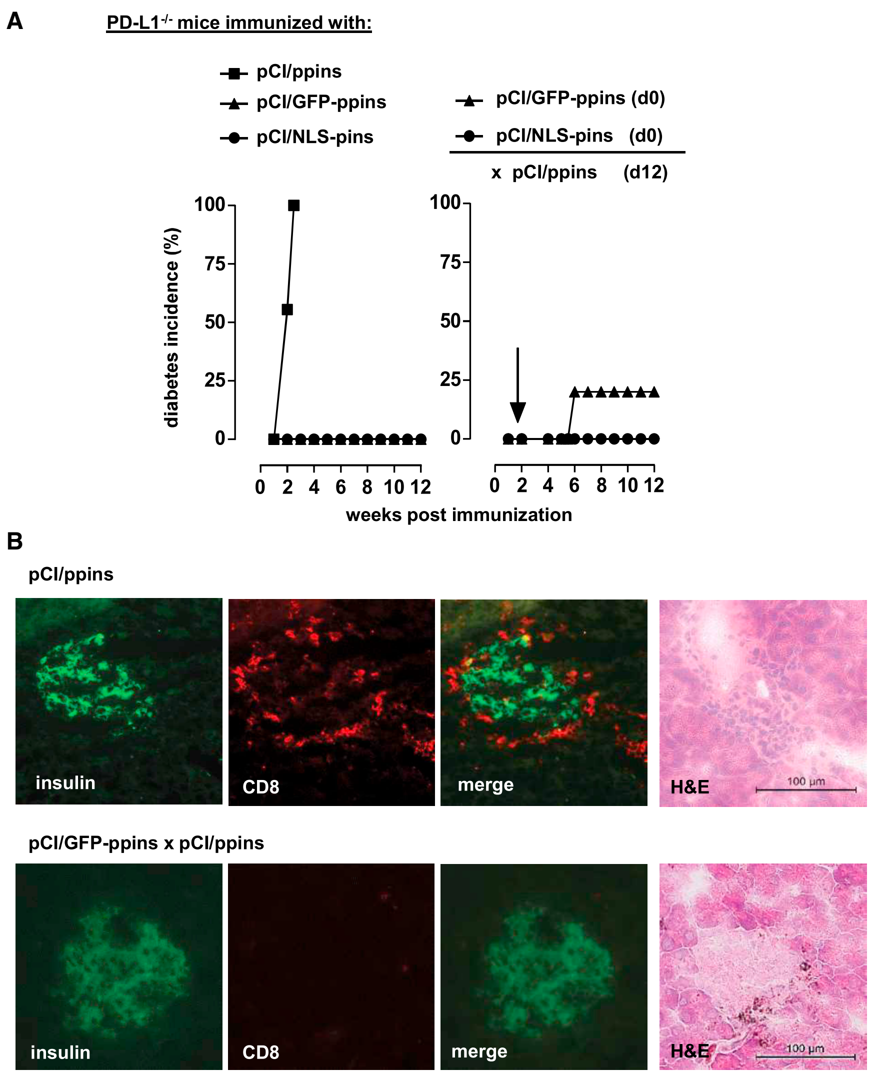

Figure 3. Determination of the Immune-Suppressive Potential of pCI/GFP-ppins and pCI/NLS-pins in *PD-L1*$^{-/-}$ Mice

(A) *PD-L1*$^{-/-}$ mice were either immunized with pCI/ppins (n = 9), pCI/GFP-ppins (n = 5), or pCI/NLS-pins alone (n = 5) (left) or immunized with pCI/GFP-ppins or pCI/NLS-pins (n = 5 per group) followed by the injection of the diabetes-inducing pCI/ppins vector at day 12 post-vaccination (right). We monitored diabetes development over 12 weeks by measuring the blood glucose values and determined the cumulative diabetes incidence (%).
(B) Pancreatic sections from diabetic (pCI/ppins) or healthy (pCI/GFP-ppins vaccinated and pCI/ppins primed) *PD-L1*$^{-/-}$ mice were analyzed by immunofluorescence staining for insulin and $CD8^+$ T cells and H&E staining. Scale bar, 100 μm.

allow *de novo* priming of autoreactive effector $CD8^+$ T cells in RIP-B7.1 tg mice by DNA immunization.

We previously showed that injection of pCI/ppins but not pCI/ppins$\Delta A_{12\text{-}21}$ DNA into H-2^b PD-1- or PD-L1-deficient mice induced $CD8^+$ T cell-mediated autoimmune diabetes (Figures S1B and S1C).[7,8] However, the failure to induce $CD8^+$ T cells in *PD-1*$^{-/-}$ and *PD-L1*$^{-/-}$ mice by pCI/ppins$\Delta A_{12\text{-}21}$ facilitated the induction of a systemic $Foxp3^+CD25^+CD4^+$ Treg cell immunity that suppressed diabetes development by *de novo* primed $K^b/A_{12\text{-}21}$-specific $CD8^+$ T cells.[18] Similarly, pCI/GFP-ppins or pCI/NLS-ppins vectors did not induce autoimmune diabetes in PD-L1-deficient mice (Figure 3A) and efficiently suppressed $CD8^+$ T-cell-mediated diabetes induction by a subsequent injection of the pCI/ppins vector (Figure 3B). In line with our previous findings, injection of pCI/GFP-ppins significantly increased $Foxp3^+CD25^+CD4^+$ Treg cell frequencies in *PD-L1*$^{-/-}$ mice (Figure S4).[18] This confirmed that ppins antigens that primarily did not induce autoreactive $CD8^+$ T cells are immunogenic and induced an immune-suppressive immunity that controls *de novo* priming and/or expansion of $K^b/A_{12\text{-}21}$-specific effector $CD8^+$ T cells in this diabetes model.[18]

Ppins or Pins Designer Antigens Excluded from Expression in the ER Suppressed Spontaneous Diabetes Development in NOD Mice

We hypothesized that ppins designer antigens that do not induce $CD8^+$ T cells and autoimmune diabetes in H-2^b RIP-B7.1 tg mice also do not induce autoreactive $CD8^+$ T cells in NOD mice, expressing the diabetes-susceptible H-2^{g7} haplotype (K^d, D^b; I-A^{g7}) but elicit a tolerogenic immunity that could suppress spontaneous diabetes development in these mice. Female NOD mice spontaneously developed severe autoimmune diabetes and hyperglycemia at the age of 14 to 22 weeks (Figure 4A). Diabetes development is characterized by continuous lymphoid cell accumulations in the periphery of pancreatic islets (peri-insulitis) and infiltration of the islets (insulitis) (Figures 5A and 5B), finally leading to the destruction of beta cells and the development of severe hyperglycemia.[20] To determine whether ppins designer antigens could induce a tolerogenic immune response in NOD mice, we injected pCI/NLS-pins or pCI/GFP-ppins vectors and different pCI-based control vectors six times in bi-weekly intervals into young female NOD mice, starting at the age of 10–12 weeks (Figures 4 and 5). As expected at this age, islets already showed signs of an initial damage with a prominent insulitis (Figure 5B, group 1),[19] whereas clinical signs of diabetes were still absent, with blood glucose levels below the threshold of 250 mg/dl (deciliter). Therefore, the destructive autoreactive immune response in the islets was already underway before DNA vaccination. In this therapeutic setting, we showed that vaccination of prediabetic

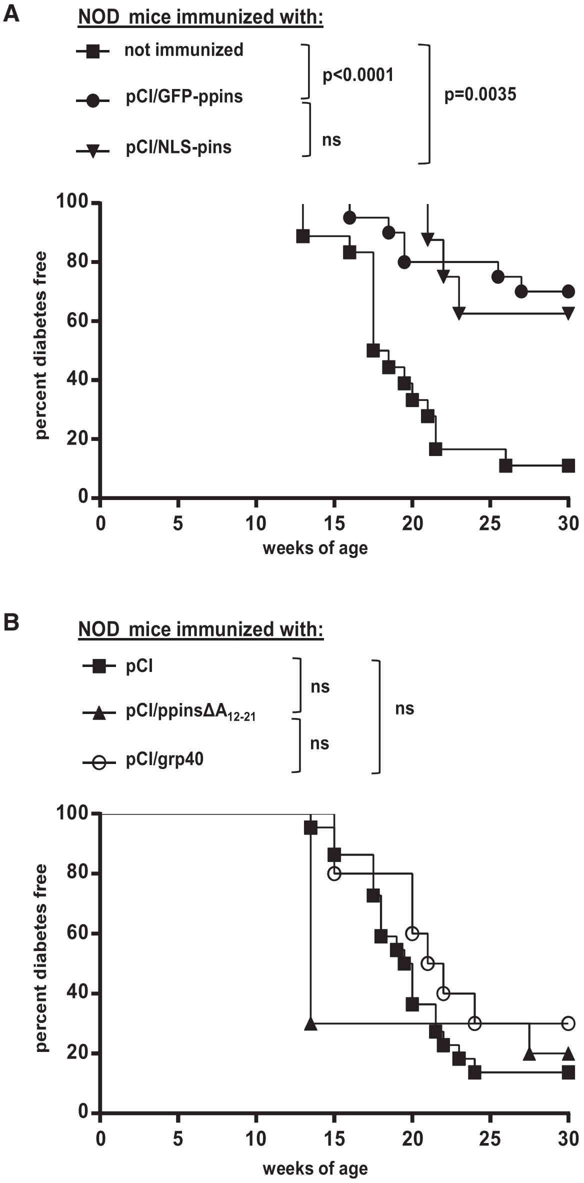

Figure 4. Suppression of Spontaneous Diabetes Development in NOD Mice by pCI/NLS-pins and pCI/GFP-ppins Vaccines

(A) Young female NOD mice, at the age of 10 to 12 weeks, were either left untreated (n = 18) or immunized with pCI/GFP-ppins (n = 20) or pCI/NLS-pins (n = 8).

(B) Furthermore, NOD mice were immunized with the "empty" pCI vector (n = 22), pCI/ppinsΔA_{12-21} (n = 10), or pCI/grp40 (n = 10). Injections were repeated six times in bi-weekly intervals. Diabetes development was monitored by regular blood glucose measurements. Kaplan-Meier curves of diabetes-free survival in the different study cohorts were compared using log-rank test. (*$p < 0.05$, **$p < 0.01$, ***$p < 0.001$); ns, not significant.

NOD mice with pCI/GFP-ppins or NLS-pins vectors significantly mitigated diabetes development as compared to untreated mice (Figure 4A). In contrast, immunization of NOD mice with pCI/ppinsΔA_{12-21} did not affect spontaneous diabetes development in

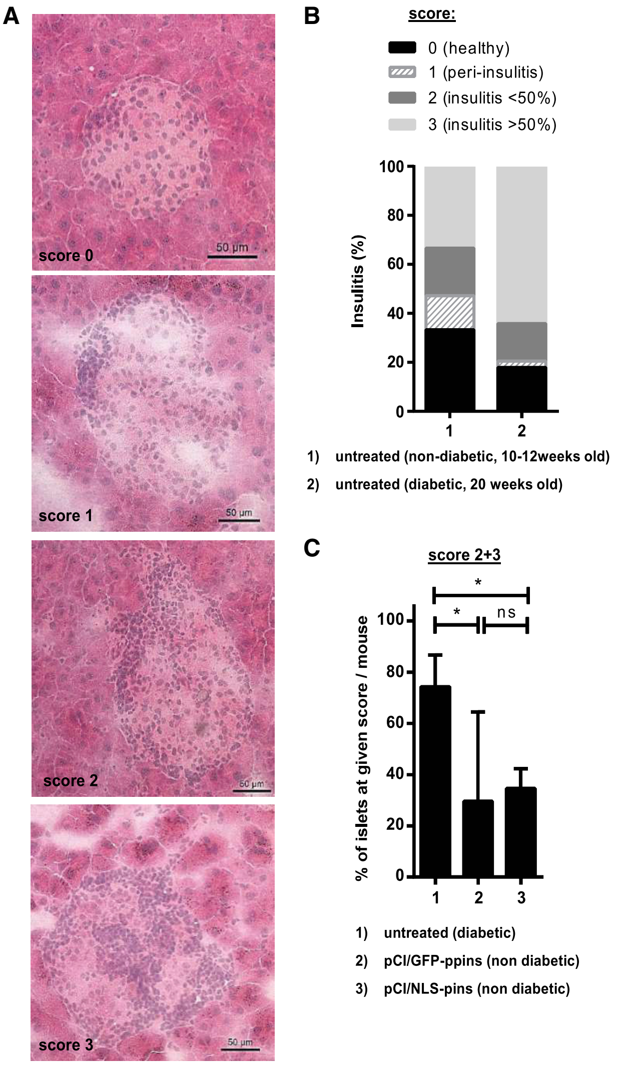

Figure 5. Suppression of the Pathogenic T Cell Infiltration into Islets of NOD Mice by pCI/NLS-pins and pCI/GFP-ppins Vaccines

(A) The continuous T cell infiltration into the islets was determined in untreated NOD mice by H&E staining of the islets. For the semiquantitative evaluation of islet infiltration, H&E-stained sections containing more than five islets were selected, and 20–30 islets per pancreas were evaluated. The extent of lymphoid islet infiltration was scored from 0 to 3 as follows: score 0 = healthy (no infiltration with lymphocytes detectable); score 1 = peri-insulitis (lymphocytes surrounding the islets); score 2 = insulitis with a <50% infiltration of the islets with lymphocytes; score 3 = severe insulitis with an extensive >50% infiltration of the islets with lymphocytes. Representative images of insulitis levels corresponding to the respective scores are shown. Scale bar, 50 μm.

(B) The actual progress of spontaneous diabetes development was determined in untreated nondiabetic (10–12 weeks old, n = 5) and diabetic (20 weeks old, n = 5) NOD mice: score 0 (black), score 1 (light gray, cross-striped), score 2 (gray), score 3 (light gray).

(C) The actual T cell infiltration of islets (insulitis scores 2 + 3) was determined in untreated diabetic (n = 5) NOD mice and compared with healthy, pCI/GFP-ppins (n = 4) or pCI/NLS-pins (n = 3) immune NOD mice. For statistical analysis, a repeated-measures one-way ANOVA followed by Tukey's pairwise comparisons was used. *$p < 0.05$; ns, not significant.

female NOD mice (Figure 4B). Likewise, injection of the "empty" pCI vector or a vector expressing the G-protein-coupled receptor GPR40 (pCI/GRP40) did not affect spontaneous diabetes development in NOD mice (Figure 4B). As compared to 20-week-old untreated, diabetic NOD mice, suppression of diabetes development in age-matched pCI/GFP-ppins- or pCI/NLS-pins-immune and healthy NOD mice correlated with a substantial reduction of lymphoid cell infiltrations (insulitis) into the islets (Figure 5C). Similarly, immunofluorescence staining of pancreata from healthy (vaccinated with pCI/GFP-ppins vector) NOD mice confirmed the perpetuation of insulin production in the islets and the little infiltration of islets with $CD8^+$ T cells and $CD4^+$ T cells (Figure S5A). In contrast, pancreatic islets of diabetic NOD mice (e.g., injected with "empty" pCI vector) showed a massive infiltration with $CD8^+$ T cells and $CD4^+$ T cells and insulin-producing beta cells were largely destroyed (Figure S5B). Overall, these findings showed that DNA vaccines expressing ppins or pins designer antigens excluded from ER efficiently inhibited an ongoing autoreactive immune response in NOD mice.

DISCUSSION

Here, we describe a novel strategy to prevent diabetes development in murine models of T1D through DNA vaccination. Vector-expressed designer antigens that targeted ppins to the cytosol and/or the nucleus and thus are excluded from direct expression and processing in the ER did not induce either autoreactive T cells or autoimmune diabetes in different mouse models, namely H-2^b (K^b, D^b, I-A^b) RIP-B7.1 tg and co-inhibition-deficient *PD-L1*$^{-/-}$ mice as well as H-2^{g7} (K^d, D^b, I-A^{g7}) NOD mice. Therefore, these vaccines are considered safe with no (or minimal) risk to induce or accelerate disease. DNA vaccines efficiently suppressed spontaneous diabetes development in female NOD mice and suppressed $CD8^+$ T cell-mediated diabetes development in co-inhibition deficient *PD-L1*$^{-/-}$ mice triggered by a single injection of pCI/ppins DNA. The vaccine-induced tolerogenic immunity was antigen (ppins or pins)-specific and could suppress multi-specific autoreactive $CD4^+$ and $CD8^+$ T cell responses in NOD mice,[19] as well as *de novo* primed ppins-specific effector $CD8^+$ T cells in *PD-L1*$^{-/-}$ mice.

Many beta cell antigens (e.g., ppins, glutamic acid decarboxylase, or islet-specific glucose-6 phosphatase catalytic subunit-related protein [IGRP]) are presented by individual MHC class I molecules on the surface of beta cells in NOD mice and man and thus are potential targets for autoreactive $CD8^+$ T cells.[19] There is increasing evidence from patients with T1D that $CD8^+$ T cells play a crucial role in the development of disease.[25–30] At least in NOD mice, ppins or insulin is the primary beta cell autoantigen targeted by autoreactive $CD4^+$ and $CD8^+$ T cells[31] and thereby triggers the initial steps of beta cell destruction and inflammation.[32] $CD8^+$ T cells specific for the islet-specific IGRP accumulate in NOD mice but not in tg NOD mice that are tolerant to pins.[33] An initial T cell response against pins is thus a prerequisite for the development of IGRP-specific $CD8^+$ T cells.[33] These early ppins-specific immune responses could subsequently drive the induction and/or expansion of multi-specific autoreactive T cell responses to other beta cell antigens.[31,33,34] However, induction and progression of autoimmune diabetes requires complex interactions between different components and/or networks of the immune system (e.g., professional APCs, $CD4^+$ and $CD8^+$ T cells, but also natural killer [NK] and natural killer T [NKT] cells). In particular, I-A^{g7}/B_{9-23} $CD4^+$ T cells could play a central role in the induction of autoimmune diabetes in NOD mice.[34,35] Therefore, the requirements for therapeutic vaccines against T1D are complex[36] and should (1) be safe and not trigger autoreactive immune responses, (2) primarily suppress autoreactive $CD4^+$ and $CD8^+$ T cell responses directed against ppins, its processing intermediates, or unusual ribosomal insulin products[37] as well as against very different beta cell antigens,[19] and (3) operate in individuals with different MHC I and II compositions. We here showed that ppins designer antigens excluded from ER expression are smart vaccination tools to fulfill these requirements.

Here, we showed that antigen expression in the ER is crucial to prime $CD8^+$ T-cell-mediated diabetes. Antigens not expressed in the ER (i.e., pCI/GFP-ppins and pCI/NLS-pins) did not induce diabetes. However, we previously showed that a pCI/pins vector, generated from pCI/ppins by removing the SP domain, expressed an instable protein in the cytosol. Comparable to the mutant ppinsΔA_{12-21} antigen, proteasome-mediated degradation of pins resulted in a high turnover of this antigen in transiently transfected cells.[6,8] Injection of pCI/pins into RIP-B7.1 tg mice inefficiently induced late-onset autoimmune diabetes,[6] but we could not unequivocally assign $CD8^+$ T cell specificities to diabetes development. Therefore, this antigen contains a residual risk to develop diabetes, though pins expressing DNA vaccines were successfully used in NOD mice[23] and patients with T1D to induce a tolerogenic immunity.[22] The ppins K^b/A_{12-21} epitope, representing the COOH terminus of the ppins molecule, does not require COOH-terminal processing for loading to K^b molecules in the ER.[6] Similarly, a B_{22-30} epitope precursor with an almost exact COOH terminus (i.e., the B_{22-30} peptide with one additional serine residue at position B30) could be generated in insulin-producing islet beta cells by beta cell-specific prohormone convertases PC2 and PC3 (cleaving between the B and C and C and A junctions, respectively) and by carboxypeptidase E (removing the COOH-terminal, basic arginine residues of the B/C junction; Figure S1B).[38] However, these enzymes were not expressed in non-beta cells (i.e., in vector-transfected APCs in the muscle).[38] The processing mechanism(s) involved in the generation of the K^b/B_{22-29} peptide in non-beta cells and its loading to K^b molecules are yet unknown, but, at least for the mutant ppinsΔA_{12-21} antigen, may depend on its instable, proteasome-mediated high turn-over expression in transfected non-beta cells.[8] In line with this, secretory or transmembrane proteins often contain MHC class I epitopes in their ER-targeting NH_2-terminal signal peptides (SPs). SPs translocate proteins into the ER and are cleaved there by ER-resident signal peptidases (SPases). This often generates MHC class I epitope(s) with an exact COOH terminus. This has been shown to occur for two overlapping HLA-A0201-binding epitopes ($ppins_{15-24}$ and $ppins_{17-24}$) in the SP of human ppins.[27] ER-resident aminopeptidase ERAP1,[39] but also signal peptide peptidases (SPPases)[40,41] can further process the NH_2-terminal

epitope-flanking sequences. Processing and presentation of these epitopes in the ER required neither proteasomes nor TAP,[27,42] thus differing from the conventional endogenous antigen-processing pathway, in which the majority of MHC class I-binding peptides are generated by the proteasome complex followed by TAP-mediated peptide translocation into the ER.[43] However, several epitopes (and $CD8^+$ T cells) were also identified in the human ppins SP that contain COOH termini far away from the natural SPase processing site at position 24/25 (HLA-A*24/$ppins_{3-11}$; HLA-B*39/$ppins_{5-12}$; HLA-B*38/$ppins_{5-14}$).[28,41] These epitopes are processed in the ER and/or the ER membrane by ER-associated SPases and SPPases, released into the cytoplasm and further processed for MHC I binding in a proteasome- and TAP-dependent manner.[28,41,44] Furthermore, it has been shown that $CD8^+$ T cells directed against an epitope of a defective ribosomal product (DRiP)[45] encoded in an alternative open reading frame of ppins are capable of killing human beta cells,[37] but the MHC I processing pathway for this antigen or epitope is unknown.

There is increasing evidence that strategies to induce $Foxp3^+CD25^+$ $CD4^+$ Treg cells may efficiently control T1D.[21,46–48] Ppins-specific DNA vaccines (e.g., encoding the ppinsΔA_{12-21}) elicited a systemic $Foxp3^+CD25^+CD4^+$ Treg cell immunity in *PD-L1*$^{-/-}$ or *PD-1*$^{-/-}$ mice that selectively suppressed $CD8^+$ T cell-mediated (and K^b/A_{12-21}-specific) diabetes induction.[18] Ablation of Treg cells in vaccinated and ppins-primed mice by anti-CD25 (PC61) antibody treatment abolished the protective effect of the vaccine and enabled diabetes induction by pCI/ppins.[18] However, we and others[23] could not unequivocally assign vaccine-primed functional regulatory Tregs to the suppression of spontaneous autoimmune diabetes in NOD mice. In our hands, injection of Treg-targeting anti-CD25 monoclonal antibody (mAb) into prediabetic NOD mice at the age of 10–12 weeks but also injection of anti-CD25 or anti-CD8 mAbs into early diabetic NOD mice (with blood glucose levels between 250 and 350 mg/dl) was inefficient and attenuated diabetes progression only in about 15%–20% of mice (data not shown). Defects in the complement system,[49] but also in many immunological pathways[50–53] in NOD mice may limit the use of these mice to elucidate the molecular mechanisms involved in vaccine-induced tolerogenic immune response.

In summary, we presented two main new findings: (1) changing the structural integrity of the vector-encoded ppins protein affected the priming of effector $CD8^+$ T cells in an epitope-specific manner; and (2) changing the natural expression of ppins in the ER prevented priming of ppins-specific $CD8^+$ T cells, suppressed the potential multi-specific immune response against beta cell antigens, and protected NOD mice from diabetes development. DNA vaccines expressing selected ppins designer antigens are thus an attractive strategy for the improvement of immune therapies against T1D.

MATERIALS AND METHODS

Mice

RIP-B7.1 mice,[3] *PD-1*$^{-/-}$ mice,[54] *PD-L1*$^{-/-}$ (*B7-H1*$^{-/-}$) mice,[55] and female NOD mice (Charles River; Calco, Italy) were bred and kept under standard pathogen-free conditions in the animal colony of Ulm University (Ulm, Germany). All mouse immunization studies were carried out in strict accordance with the recommendations in the Guide for the Care and Use of Laboratory Animals of the German Federal Animal Protection Law. The protocols were approved by the Committee on the Ethics of Animal Experiments of the University of Ulm (Tierforschungszentrum Ulm, Oberberghof) and the Regierungspräsidium Tübingen (permit numbers 1105, 1199, and 1327 to R.S.). Immunizations were performed under short-time Isofluran anesthesia, and all efforts were made to minimize suffering.

Construction of Expression Plasmids

The antigenic sequences of the ppins variants were codon optimized and synthesized by GeneArt (Regensburg, Germany) or generated from these constructs by standard cloning techniques. All constructs were cloned into the pCI vector (cat. no. E1731, Promega, Mannheim, Germany) using the *NheI* and *NotI* restriction sites. Batches of DNA were produced in *E. coli* using the QIAGEN Plasmid Mega Kit (cat. no. 12183; QIAGEN, Hilden, Germany).

Immunization of Mice and Detection of Ppins-Specific $CD8^+$ T Cells

Mice were immunized into both tibialis anterior muscles with 100 μg/mouse of plasmid DNA. Development of autoimmune diabetes was analyzed by regular blood glucose measurements and diagnosed if two consecutive blood glucose values (within 2 days) exceeded 250 mg/dl, i.e., 13.8 mmol/L (Disetronic Freestyle, Sulzbach, Germany). A single drop of blood for the measurements was obtained by tail-vein puncture.

K^b/B_{22-29}-specific $CD8^+$ T cell frequencies were determined in the pancreata as described previously.[8,12] K^b/B_{22-29}-specific $CD8^+$ T cells were stained with APC-conjugated anti-CD8 mAb (cat. no. 17-0081-83, BD Biosciences) and phycoerythrin (PE)-conjugated $K^b/B_{22-M29L}$ loaded MHC class I dimers (BDDimer X, cat. no. 552944, BD Biosciences).

Characterization of Antigen Expression

HEK293 cells (ATCC CRL-1573) were used to determine expression of ppins constructs, because they can be transfected with high efficacy ($\geq$90%) using the calcium phosphate method and express high levels of vector-encoded antigens.[56] For western blot analyses, transiently transfected cells were directly lysed with SDS-containing buffer (50 mM Tris-hydrochloride, 3% SDS, 5% β-mercaptoethanol [pH 6.8]), processed for SDS-PAGE and blotted on nitrocellulose membranes (cat. no. IB3010-01, Thermo Fisher, Germany) using the iBlot Dry Blotting system (Thermo Fisher). Membranes were blocked for 30 min at room temperature (RT) in a buffer supplemented with 0.1% Tween 20, 0.1% gelatin, and 3% milk powder. Membranes were successively incubated with rabbit H-86 anti-insulin antibody (cat. no. sc-9168, Santa Cruz Biotechnology) and horseradish peroxidase (HRP)-conjugated anti-rabbit immunoglobulin G (IgG) (cat. no. NA9340; GE Healthcare, Chalfont St Giles, UK). Where indicated, membranes were incubated with Restore Western Blot Stripping

Buffer (cat. no. 21059; Thermo Fisher Scientific) according to the recommendations of the manufacturer, prior to incubation with mouse anti-beta-actin mAb (cat. no. A2228, Sigma Munich, Germany) and HRP-conjugated sheep anti-mouse IgG (cat. no. NA931V, GE Healthcare, Dornstadt, Germany). HA-tagged proteins were detected using a primary anti-HA-Tag antibody (6E2, cat. no. 2367, Cell Signaling, Denver, MA, USA) and the secondary HRP-conjugated sheep anti-mouse antibody. The membranes were dried, and the Immobilon Western Chemoluminescent HRP substrate was applied as recommended by the manufacturer (cat. no. WBKLS0100, Millipore, Darmstadt, Germany) followed by exposure of the membranes to an Amersham Hyperfilm ECL (cat. no. 28906847, GE Healthcare, Dornstadt, Germany).

Immunofluorescence Staining

HeLa cells were grown in 2 mL of medium (cat. no. 31885, Invitrogen) supplemented with 10% fetal calf serum (FCS) on fibronectin (cat. no. 10838039001, Roche)-coated glass coverslips.[8,18] Where indicated, cells were co-transfected with vectors encoding the L-RFP-ER protein (the red fluorescent protein [RFP] fused NH_2-terminally with a Igκ-leader sequence and COOH terminally with the ER-retention signal SEKDEL) or encoding a red fluorescent mRuby-histone 2B protein using the Nanofectin transfection reagent (cat. no. Q051/005, PAA Laboratories, Cölbe, Germany). For co-localization studies, cells were transfected with the indicated plasmids, fixed with 2% paraformaldehyde (PFA), and permeabilized with 0.2% Triton X-100, 48 hr post-transfection. Fish-skin gelatin (0.2%) was used as blocking reagent. Cells were stained for expression of the recombinant proteins, using rabbit H-86 anti-insulin antibody or mouse anti-HA-Tag antibody (6E2, cat. no. 2367, Cell Signaling, Denver, MA, USA), followed by Alexa Fluor 488-conjugated goat anti-rabbit IgG (cat. no. ab150077, Abcam, Cambridge, UK) or Alexa Fluor 488-conjugated goat anti-mouse IgG (cat. no. ab150113, Abcam), respectively. Images were acquired with a fluorescence microscope (IX71; Olympus) equipped with a digital camera (C4742; Hamamatsu), a 100-W mercury lamp (HBO 103W/2; Osram), and the following filter sets: GFP, excitation HQ470/40, emission HQ525/50; RFP, excitation HQ545/30, emission HQ610/75 (AHF Analysentechnik). Editing of the pictures was performed using ImageJ software (https://imagej.nih.gov/if/).

Histology

H&E and immunofluorescence staining of frozen pancreatic sections was performed as described previously.[8,18] For immunofluorescence staining, the following antibodies were used: polyclonal guinea pig anti-insulin serum (cat. no. A0564; Dako, Carpinteria, CA, USA), rat anti-CD8 (cat. no. MCA2694; AbD Serotec, Oxford, UK), or rat anti-CD4 (cat. no. MCA1767GA; AbD Serotec), anti-guinea pig IgG-fluorescein isothiocyanate (FITC) (cat. no. F-6261; Sigma-Aldrich, St. Louis, MO, USA) and anti-rat IgG-TRITC (cat. no. T4280; Sigma-Aldrich). Sections were covered with Cytoseal60 mounting medium (cat. no. 18006, Electron Microscopy Sciences, Hatfield, PA, USA). Images were captured with an Olympus IX71 fluorescence microscope equipped with a digital camera (C4742, Hamamatsu). Editing of the pictures was performed using ImageJ software (https://imagej.nih.gov/if/). Images of H&E-stained sections were acquired on a light microscope (Leica, Germany) equipped with a digital camera and Leica Application Suite software (Leica Microsystems, Switzerland).

Statistical Analysis

PRISM 6.04 GraphPad software (GraphPad, San Diego, CA, USA) was used for statistical analyses. Figures show mean values ± SD, and group sizes are stated in the figure descriptions. For the evaluation of statistical differences in the mean T cell frequencies between groups and differences between insulitis grades of the differently treated NOD study cohorts, one-way ANOVA followed by Tukey's multiple comparisons (with 95% confidence intervals) was used. (* $p < 0.05$, ** $p < 0.01$, *** $p < 0.001$) The statistical significance of diabetes induction in immunized female NOD mice was determined by the log-rank (Mantel-Cox) test.

SUPPLEMENTAL INFORMATION

Supplemental Information includes five figures and can be found with this article online at https://doi.org/10.1016/j.omtm.2018.12.002.

AUTHOR CONTRIBUTIONS

K.S., C.S., J.K., and A.S. performed the experiments, researched data, and contributed to discussion; B.O.B. contributed to discussion and reviewed/edited the manuscript; K.S. and R.S. conceived and designed the experiments and wrote the manuscript.

CONFLICTS OF INTEREST

The authors have no conflicting financial interests.

ACKNOWLEDGMENTS

We thank Kathrin Schwengle for outstanding technical assistance. We thank Dr. Franz Oswald (University Hospital Ulm, Germany) for L-RFP-ER and mRuby-H2B vectors. We thank Dr. T. Honjo (Department of Immunology and Genomic Medicine, Kyoto University, Kyoto, Japan) for *PD-1*$^{-/-}$ mice and Dr. Lieping Chen (Department of Oncology, Johns Hopkins University School of Medicine, Baltimore, MD, USA) for *PD-L1 (B7-H1)*$^{-/-}$ mice. This work was supported by grants from the Deutsche Forschungsgemeinschaft: GRK-1789 "CEMMA" and DFG SCHI-505/6-1 to R.S. B.O.B. is supported by Lee Kong Chian School of Medicine, Nanyang Technological University Start Up Grant, Ministry of Education - Singapore (MOE), OE AcRF Tier 1 (2015-T1-001-258), and a MOE Tier 1 grant.

REFERENCES

1. Garren, H. (2009). DNA vaccines for autoimmune diseases. Expert Rev. Vaccines *8*, 1195–1203.
2. Johnson, M.C., Wang, B., and Tisch, R. (2011). Genetic vaccination for re-establishing T-cell tolerance in type 1 diabetes. Hum. Vaccin. *7*, 27–36.
3. Harlan, D.M., Hengartner, H., Huang, M.L., Kang, Y.H., Abe, R., Moreadith, R.W., Pircher, H., Gray, G.S., Ohashi, P.S., Freeman, G.J., et al. (1994). Mice expressing both B7-1 and viral glycoprotein on pancreatic beta cells along with glycoprotein-specific transgenic T cells develop diabetes due to a breakdown of T-lymphocyte unresponsiveness. Proc. Natl. Acad. Sci. USA *91*, 3137–3141.

4. Karges, W., Pechhold, K., Al Dahouk, S., Riegger, I., Rief, M., Wissmann, A., Schirmbeck, R., Barth, C., and Boehm, B.O. (2002). Induction of autoimmune diabetes through insulin (but not GAD65) DNA vaccination in nonobese diabetic and in RIP-B7.1 mice. Diabetes *51*, 3237–3244.

5. Karges, W., Rajasalu, T., Spyrantis, A., Wieland, A., Boehm, B., and Schirmbeck, R. (2007). The diabetogenic, insulin-specific CD8 T cell response primed in the experimental autoimmune diabetes model in RIP-B7.1 mice. Eur. J. Immunol. *37*, 2097–2103.

6. Brosi, H., Reiser, M., Rajasalu, T., Spyrantis, A., Oswald, F., Boehm, B.O., and Schirmbeck, R. (2009). Processing in the endoplasmic reticulum generates an epitope on the insulin A chain that stimulates diabetogenic CD8 T cell responses. J. Immunol. *183*, 7187–7195.

7. Rajasalu, T., Brosi, H., Schuster, C., Spyrantis, A., Boehm, B.O., Chen, L., Reimann, J., and Schirmbeck, R. (2010). Deficiency in B7-H1 (PD-L1)/PD-1 coinhibition triggers pancreatic beta-cell destruction by insulin-specific, murine CD8 T-cells. Diabetes *59*, 1966–1973.

8. Schuster, C., Brosi, H., Stifter, K., Boehm, B.O., and Schirmbeck, R. (2013). A missing PD-L1/PD-1 coinhibition regulates diabetes induction by preproinsulin-specific CD8 T-cells in an epitope-specific manner. PLoS ONE *8*, e71746.

9. Fuchs, Y.F., Adler, K., Lindner, A., Karasinsky, A., Wilhelm, C., Weigelt, M., Balke, H., Förtsch, K., Mortler-Hildebrandt, L.F., Harlan, D.M., et al. (2014). IGRP and insulin vaccination induce CD8+ T cell-mediated autoimmune diabetes in the RIP-CD80GP mouse. Clin. Exp. Immunol. *176*, 199–206.

10. Verhagen, J., Smith, E.L., Whettlock, E.M., Macintyre, B., and Peakman, M. (2018). Proinsulin-mediated induction of type 1 diabetes in HLA-DR4-transgenic mice. Sci. Rep. *8*, 14106.

11. Pechhold, K., Karges, W., Blum, C., Boehm, B.O., and Harlan, D.M. (2003). Beta cell-specific CD80 (B7-1) expression disrupts tissue protection from autoantigen-specific CTL-mediated diabetes. J. Autoimmun. *20*, 1–13.

12. Redmond, W.L., and Sherman, L.A. (2005). Peripheral tolerance of CD8 T lymphocytes. Immunity *22*, 275–284.

13. Zehn, D., and Bevan, M.J. (2006). T cells with low avidity for a tissue-restricted antigen routinely evade central and peripheral tolerance and cause autoimmunity. Immunity *25*, 261–270.

14. Zehn, D., Lee, S.Y., and Bevan, M.J. (2009). Complete but curtailed T-cell response to very low-affinity antigen. Nature *458*, 211–214.

15. Stadinski, B.D., Zhang, L., Crawford, F., Marrack, P., Eisenbarth, G.S., and Kappler, J.W. (2010). Diabetogenic T cells recognize insulin bound to IAg7 in an unexpected, weakly binding register. Proc. Natl. Acad. Sci. USA *107*, 10978–10983.

16. Mohan, J.F., Petzold, S.J., and Unanue, E.R. (2011). Register shifting of an insulin peptide-MHC complex allows diabetogenic T cells to escape thymic deletion. J. Exp. Med. *208*, 2375–2383.

17. Hsu, H.T., Janßen, L., Lawand, M., Kim, J., Perez-Arroyo, A., Culina, S., Gdoura, A., Burgevin, A., Cumenal, D., Fourneau, Y., et al. (2014). Endoplasmic reticulum targeting alters regulation of expression and antigen presentation of proinsulin. J. Immunol. *192*, 4957–4966.

18. Stifter, K., Schuster, C., Schlosser, M., Boehm, B.O., and Schirmbeck, R. (2016). Exploring the induction of preproinsulin-specific Foxp3(+) CD4(+) Treg cells that inhibit CD8(+) T cell-mediated autoimmune diabetes by DNA vaccination. Sci. Rep. *6*, 29419.

19. Chen, Y.G., Mathews, C.E., and Driver, J.P. (2018). The Role of NOD Mice in Type 1 Diabetes Research: Lessons from the Past and Recommendations for the Future. Front. Endocrinol. (Lausanne) *9*, 51.

20. Regnell, S.E., and Lernmark, Å. (2017). Early prediction of autoimmune (type 1) diabetes. Diabetologia *60*, 1370–1381.

21. Alhadj Ali, M., Liu, Y.F., Arif, S., Tatovic, D., Shariff, H., Gibson, V.B., Yusuf, N., Baptista, R., Eichmann, M., Petrov, N., et al. (2017). Metabolic and immune effects of immunotherapy with proinsulin peptide in human new-onset type 1 diabetes. Sci. Transl. Med. *9*, eaaf7779.

22. Roep, B.O., Solvason, N., Gottlieb, P.A., Abreu, J.R.F., Harrison, L.C., Eisenbarth, G.S., Yu, L., Leviten, M., Hagopian, W.A., Buse, J.B., et al.; BHT-3021 Investigators (2013). Plasmid-encoded proinsulin preserves C-peptide while specifically reducing proinsulin-specific $CD8^+$ T cells in type 1 diabetes. Sci. Transl. Med. *5*, 191ra82.

23. Solvason, N., Lou, Y.P., Peters, W., Evans, E., Martinez, J., Ramirez, U., Ocampo, A., Yun, R., Ahmad, S., Liu, E., et al. (2008). Improved efficacy of a tolerizing DNA vaccine for reversal of hyperglycemia through enhancement of gene expression and localization to intracellular sites. J. Immunol. *181*, 8298–8307.

24. Schirmbeck, R., Kwissa, M., Fissolo, N., Elkholy, S., Riedl, P., and Reimann, J. (2002). Priming polyvalent immunity by DNA vaccines expressing chimeric antigens with a stress protein-capturing, viral J-domain. FASEB J. *16*, 1108–1110.

25. Pinkse, G.G., Tysma, O.H., Bergen, C.A., Kester, M.G., Ossendorp, F., van Veelen, P.A., Keymeulen, B., Pipeleers, D., Drijfhout, J.W., and Roep, B.O. (2005). Autoreactive CD8 T cells associated with beta cell destruction in type 1 diabetes. Proc. Natl. Acad. Sci. USA *102*, 18425–18430.

26. Mallone, R., Martinuzzi, E., Blancou, P., Novelli, G., Afonso, G., Dolz, M., Bruno, G., Chaillous, L., Chatenoud, L., Bach, J.M., and van Endert, P. (2007). CD8+ T-cell responses identify beta-cell autoimmunity in human type 1 diabetes. Diabetes *56*, 613–621.

27. Skowera, A., Ellis, R.J., Varela-Calviño, R., Arif, S., Huang, G.C., Van-Krinks, C., Zaremba, A., Rackham, C., Allen, J.S., Tree, T.I., et al. (2008). CTLs are targeted to kill beta cells in patients with type 1 diabetes through recognition of a glucose-regulated preproinsulin epitope. J. Clin. Invest. *118*, 3390–3402.

28. Kronenberg, D., Knight, R.R., Estorninho, M., Ellis, R.J., Kester, M.G., de Ru, A., Eichmann, M., Huang, G.C., Powrie, J., Dayan, C.M., et al. (2012). Circulating preproinsulin signal peptide-specific CD8 T cells restricted by the susceptibility molecule HLA-A24 are expanded at onset of type 1 diabetes and kill β-cells. Diabetes *61*, 1752–1759.

29. Abreu, J.R., and Roep, B.O. (2013). Targeting proinsulin-reactive CD8+ T cells: a new direction for type 1 diabetes treatment. Expert Rev. Clin. Immunol. *9*, 1001–1003.

30. Yeo, L., Woodwyk, A., Sood, S., Lorenc, A., Eichmann, M., Pujol-Autonell, I., Melchiotti, R., Skowera, A., Fidanis, E., Dolton, G.M., et al. (2018). Autoreactive T effector memory differentiation mirrors β cell function in type 1 diabetes. J. Clin. Invest. *128*, 3460–3474.

31. Nakayama, M., Abiru, N., Moriyama, H., Babaya, N., Liu, E., Miao, D., Yu, L., Wegmann, D.R., Hutton, J.C., Elliott, J.F., and Eisenbarth, G.S. (2005). Prime role for an insulin epitope in the development of type 1 diabetes in NOD mice. Nature *435*, 220–223.

32. Wong, F.S., Siew, L.K., and Wen, L. (2008). CD8+ T-cells and their interaction with other cells in damage to islet beta-cells. Biochem. Soc. Trans. *36*, 316–320.

33. Krishnamurthy, B., Dudek, N.L., McKenzie, M.D., Purcell, A.W., Brooks, A.G., Gellert, S., Colman, P.G., Harrison, L.C., Lew, A.M., Thomas, H.E., and Kay, T.W. (2006). Responses against islet antigens in NOD mice are prevented by tolerance to proinsulin but not IGRP. J. Clin. Invest. *116*, 3258–3265.

34. Prasad, S., Kohm, A.P., McMahon, J.S., Luo, X., and Miller, S.D. (2012). Pathogenesis of NOD diabetes is initiated by reactivity to the insulin B chain 9-23 epitope and involves functional epitope spreading. J. Autoimmun. *39*, 347–353.

35. Daniel, C., Weigmann, B., Bronson, R., and von Boehmer, H. (2011). Prevention of type 1 diabetes in mice by tolerogenic vaccination with a strong agonist insulin mimetope. J. Exp. Med. *208*, 1501–1510.

36. Cook, D.P., Gysemans, C., and Mathieu, C. (2017). Prospects of a type 1 diabetes vaccine. Expert Opin. Biol. Ther. *17*, 403–406.

37. Kracht, M.J., van Lummel, M., Nikolic, T., Joosten, A.M., Laban, S., van der Slik, A.R., van Veelen, P.A., Carlotti, F., de Koning, E.J., Hoeben, R.C., et al. (2017). Autoimmunity against a defective ribosomal insulin gene product in type 1 diabetes. Nat. Med. *23*, 501–507.

38. Smeekens, S.P., Montag, A.G., Thomas, G., Albiges-Rizo, C., Carroll, R., Benig, M., Phillips, L.A., Martin, S., Ohagi, S., Gardner, P., et al. (1992). Proinsulin processing by the subtilisin-related proprotein convertases furin, PC2, and PC3. Proc. Natl. Acad. Sci. USA *89*, 8822–8826.

39. Saveanu, L., Carroll, O., Lindo, V., Del Val, M., Lopez, D., Lepelletier, Y., Greer, F., Schomburg, L., Fruci, D., Niedermann, G., and van Endert, P.M. (2005). Concerted peptide trimming by human ERAP1 and ERAP2 aminopeptidase complexes in the endoplasmic reticulum. Nat. Immunol. *6*, 689–697.

40. Voss, M., Schröder, B., and Fluhrer, R. (2013). Mechanism, specificity, and physiology of signal peptide peptidase (SPP) and SPP-like proteases. Biochim. Biophys. Acta *1828*, 2828–2839.

41. Kronenberg-Versteeg, D., Eichmann, M., Russell, M.A., de Ru, A., Hehn, B., Yusuf, N., van Veelen, P.A., Richardson, S.J., Morgan, N.G., Lemberg, M.K., and Peakman, M. (2018). Molecular Pathways for Immune Recognition of Preproinsulin Signal Peptide in Type 1 Diabetes. Diabetes *67*, 687–696.

42. El Hage, F., Stroobant, V., Vergnon, I., Baurain, J.F., Echchakir, H., Lazar, V., Chouaib, S., Coulie, P.G., and Mami-Chouaib, F. (2008). Preprocalcitonin signal peptide generates a cytotoxic T lymphocyte-defined tumor epitope processed by a proteasome-independent pathway. Proc. Natl. Acad. Sci. USA *105*, 10119–10124.

43. Oliveira, C.C., and van Hall, T. (2015). Alternative Antigen Processing for MHC Class I: Multiple Roads Lead to Rome. Front. Immunol. *6*, 298.

44. Bland, F.A., Lemberg, M.K., McMichael, A.J., Martoglio, B., and Braud, V.M. (2003). Requirement of the proteasome for the trimming of signal peptide-derived epitopes presented by the nonclassical major histocompatibility complex class I molecule HLA-E. J. Biol. Chem. *278*, 33747–33752.

45. Antón, L.C., and Yewdell, J.W. (2014). Translating DRiPs: MHC class I immunosurveillance of pathogens and tumors. J. Leukoc. Biol. *95*, 551–562.

46. Gibson, V.B., Nikolic, T., Pearce, V.Q., Demengeot, J., Roep, B.O., and Peakman, M. (2015). Proinsulin multi-peptide immunotherapy induces antigen-specific regulatory T cells and limits autoimmunity in a humanized model. Clin. Exp. Immunol. *182*, 251–260.

47. Bluestone, J.A., Buckner, J.H., Fitch, M., Gitelman, S.E., Gupta, S., Hellerstein, M.K., Herold, K.C., Lares, A., Lee, M.R., Li, K., et al. (2015). Type 1 diabetes immunotherapy using polyclonal regulatory T cells. Sci. Transl. Med. *7*, 315ra189.

48. Patterson, S.J., Pesenacker, A.M., Wang, A.Y., Gillies, J., Mojibian, M., Morishita, K., Tan, R., Kieffer, T.J., Verchere, C.B., Panagiotopoulos, C., and Levings, M.K. (2016). T regulatory cell chemokine production mediates pathogenic T cell attraction and suppression. J. Clin. Invest. *126*, 1039–1051.

49. Baxter, A.G., and Cooke, A. (1993). Complement lytic activity has no role in the pathogenesis of autoimmune diabetes in NOD mice. Diabetes *42*, 1574–1578.

50. Serreze, D.V., Gaskins, H.R., and Leiter, E.H. (1993). Defects in the differentiation and function of antigen presenting cells in NOD/Lt mice. J. Immunol. *150*, 2534–2543.

51. Kukreja, A., Costi, G., Marker, J., Zhang, C.H., Sinha, S., Sun, Z., and Maclaren, N. (2002). NKT cell defects in NOD mice suggest therapeutic opportunities. J. Autoimmun. *19*, 117–128.

52. Cotta-de-Almeida, V., Villa-Verde, D.M., Lepault, F., Pléau, J.M., Dardenne, M., and Savino, W. (2004). Impaired migration of NOD mouse thymocytes: a fibronectin receptor-related defect. Eur. J. Immunol. *34*, 1578–1587.

53. Suwanai, H., Wilcox, M.A., Mathis, D., and Benoist, C. (2010). A defective Il15 allele underlies the deficiency in natural killer cell activity in nonobese diabetic mice. Proc. Natl. Acad. Sci. USA *107*, 9305–9310.

54. Nishimura, H., Minato, N., Nakano, T., and Honjo, T. (1998). Immunological studies on PD-1 deficient mice: implication of PD-1 as a negative regulator for B cell responses. Int. Immunol. *10*, 1563–1572.

55. Dong, H., Zhu, G., Tamada, K., Flies, D.B., van Deursen, J.M., and Chen, L. (2004). B7-H1 determines accumulation and deletion of intrahepatic CD8(+) T lymphocytes. Immunity *20*, 327–336.

56. Wieland, A., Denzel, M., Schmidt, E., Kochanek, S., Kreppel, F., Reimann, J., and Schirmbeck, R. (2008). Recombinant complexes of antigen with stress proteins are potent CD8 T-cell-stimulating immunogens. J. Mol. Med. (Berl.) *86*, 1067–1079.

OMTM, Volume *12*

Supplemental Information

Preproinsulin Designer Antigens Excluded from Endoplasmic Reticulum Suppressed Diabetes Development in NOD Mice by DNA Vaccination

Katja Stifter, Cornelia Schuster, Jana Krieger, Andreas Spyrantis, Bernhard Otto Boehm, and Reinhold Schirmbeck

a

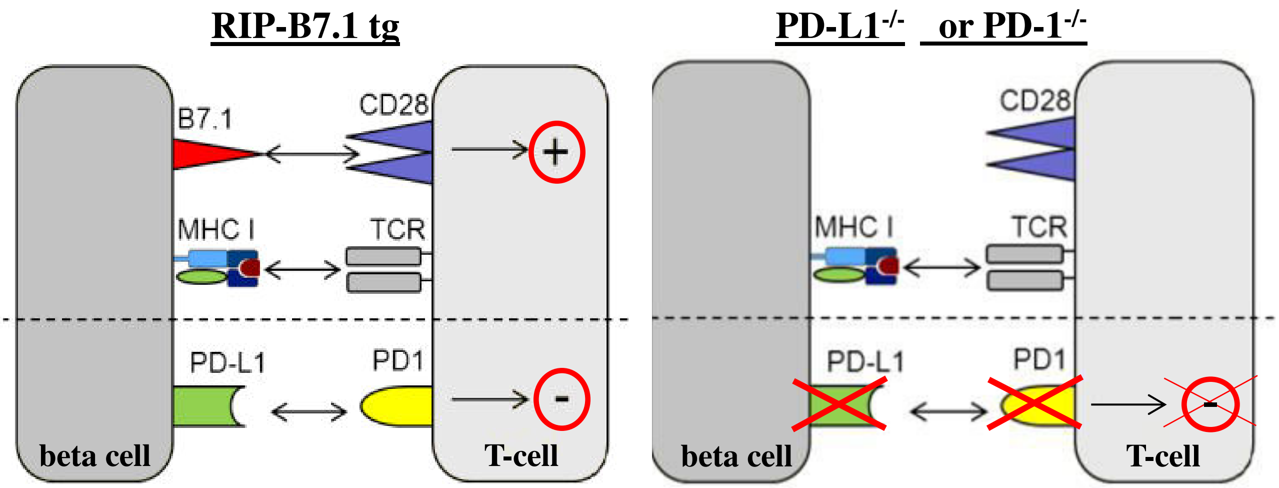

b

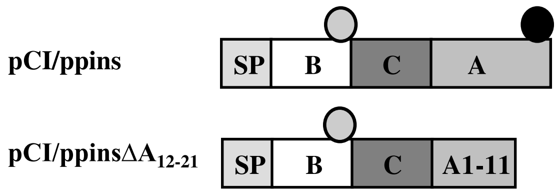

SP 1-24:ppins $_{1\text{-}24}$ B 1-30:ppins $_{25\text{-}54}$ *RR* C 1-31:ppins $_{57\text{-}87}$ *KR* A 1-21:ppins $_{90\text{-}110}$

● **K^b/A12-21 : SLYQLENYCN (ppins 101-110)**
○ **K^b/B22-29: RGFFYTPM (ppins 46-53)**

c

RIP-B7.1 tg	K^b/A$_{12-21}$	K^b/B$_{22-29}$	diabetes
ppins	+	-	+
ppinsΔA$_{12-21}$	-	+	+

PD-1/PD-L1$^{-/-}$	K^b/A$_{12-21}$	K^b/B$_{22-29}$	diabetes
ppins	+	-	+
ppinsΔA$_{12-21}$	-	-	-

Supplementary Fig. S1

Diabetes induction in the RIP-B7.1 tg mouse model. (a) Schematic presentation of the beta cell and T cell interaction in RIP-B7.1 tg, PD-L1$^{-/-}$ and PD-1$^{-/-}$ mice. (b) Map of the pCI-encoded ppins- and ppinsΔA$_{12-21}$-antigens. The positions of the insulin signal peptide (SP), B-, C- and A-chain, as well as the position and sequences of the two K^b-restricted epitopes (K^b/A$_{12-21}$ and K^b/B$_{22-29}$) are indicated. (c) Diabetes induction in RIP-B7.1 tg mice after pCI/ppins immunization is driven by K^b/A$_{12-21}$-monospecific CD8$^+$ T cells, while pCI/ppinsΔA$_{12-21}$-injection exclusively leads to induction of K^b/B$_{22-29}$-specific CD8$^+$ T cells and diabetes. In PD-L1$^{-/-}$ and PD-1$^{-/-}$ mice only pCI/ppins-immunization induces a K^b/A$_{12-21}$-specific diabetogenic CD8$^+$ T cell response, while pCI/ppinsΔA$_{12-21}$-injection induces neither K^b/B$_{22-29}$-specific CD8$^+$ T cells nor diabetes.

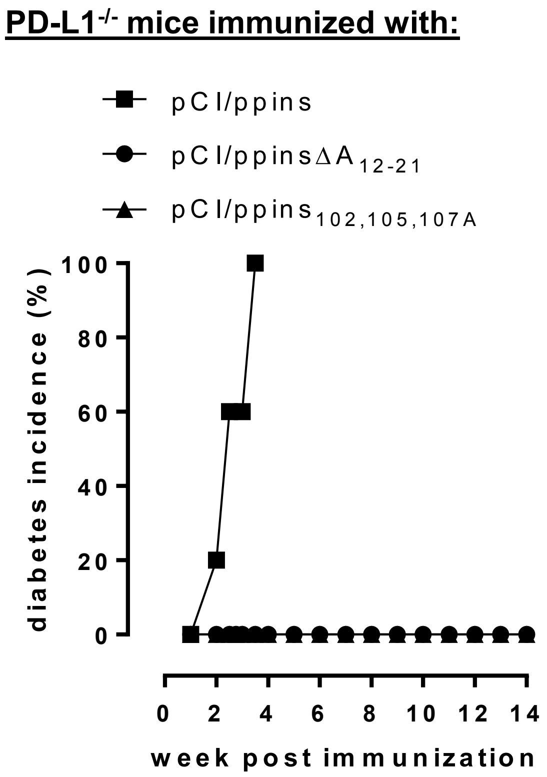

Supplementary Fig. S2
Diabetes suppressive potential of pCI/ppins$\Delta A_{12\text{-}21}$ and pCI/ppins$_{102,105,107A}$ in PD-L1$^{-/-}$ mice. PD-L1$^{-/-}$ mice were immunized with pCI/ppins, pCI/ppins$\Delta A_{12\text{-}21}$ or pCI/ppins$_{102,105,107A}$ (n=5 per group) and diabetes development was followed by regular blood glucose measurements and determination of the diabetes incidence (%).

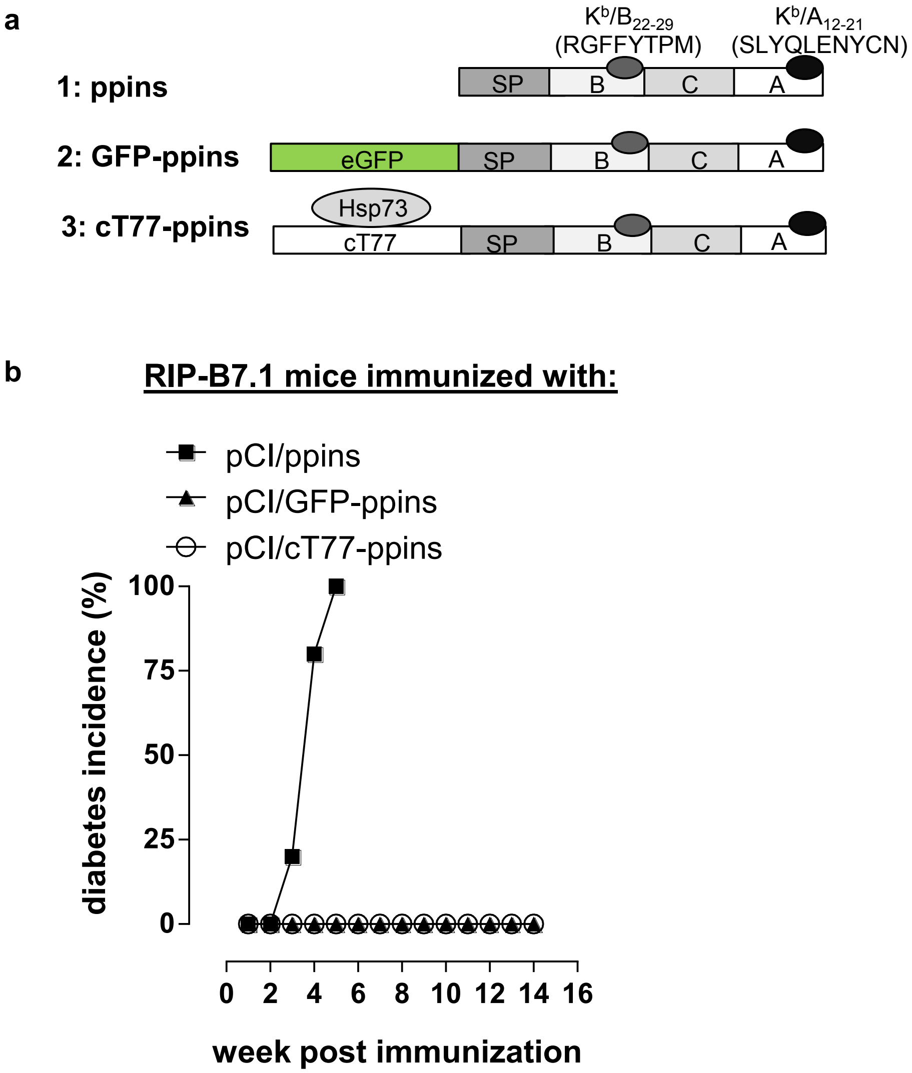

Supplementary Fig. S3

Evaluation of the diabetogenic potential of ppins-designer antigens GFP-ppins and cT77-ppins in RIP-B7.1 tg mice. (a) Maps of ppins and the designer antigens GFP-ppins and cT77-ppins. The positions and aa sequences of the ppins epitopes K^b/A_{12-21} and K^b/B_{22-29} are shown. The binding of Hsp73 to the cT77-domain (77 aa fragment of the SV40 large T-antigen) is indicated. (b) Diabetes development after pCI/GFP-ppins- or pCI/cT77-ppins-immunization of RIP-B7.1 tg mice, compared to pCI/ppins-immunization (n=5 per group), was followed by regular determination of the blood glucose levels and cumulative diabetes incidences (%).

PD-L1$^{-/-}$ mice immunized with:

1) pCI
2) pCI/GFP-ppins (d0, d12)
3) pCI/GFP-ppins (d0), pCI/ppins (d12)

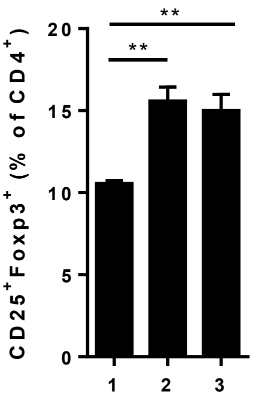

Supplementary Fig. S4
Induction of Foxp3$^+$CD25$^+$CD4$^+$ Treg cells in PD-L1$^{-/-}$ mice by pCI/GFP-ppins-vaccination. PD-L1$^{-/-}$ mice were sham-immunized (pCI, group 1, n=5), vaccinated twice (on day 0 and day 12) with pCI/GFP-ppins (group 2, n=4), or vaccinated with pCI/GFP-ppins followed by pCI/ppins-priming at day 12 post vaccination (group 3, n=4). On day 14 post pCI/ppins-injection, lymphocytes derived from pancreatic lymph nodes were stained for CD25$^+$Foxp3$^+$ regulatory CD4$^+$ T cells. Bar graphs show the frequencies of CD25$^+$Foxp3$^+$ cells among the CD4$^+$ T cell population + SD. Indicated statistically significant differences between groups 1 and 2 and between groups 1 and 3 were determined using the unpaired student's t-test. A value of (*) $p < 0.05$ was considered significant (** significant at $p < 0.01$).

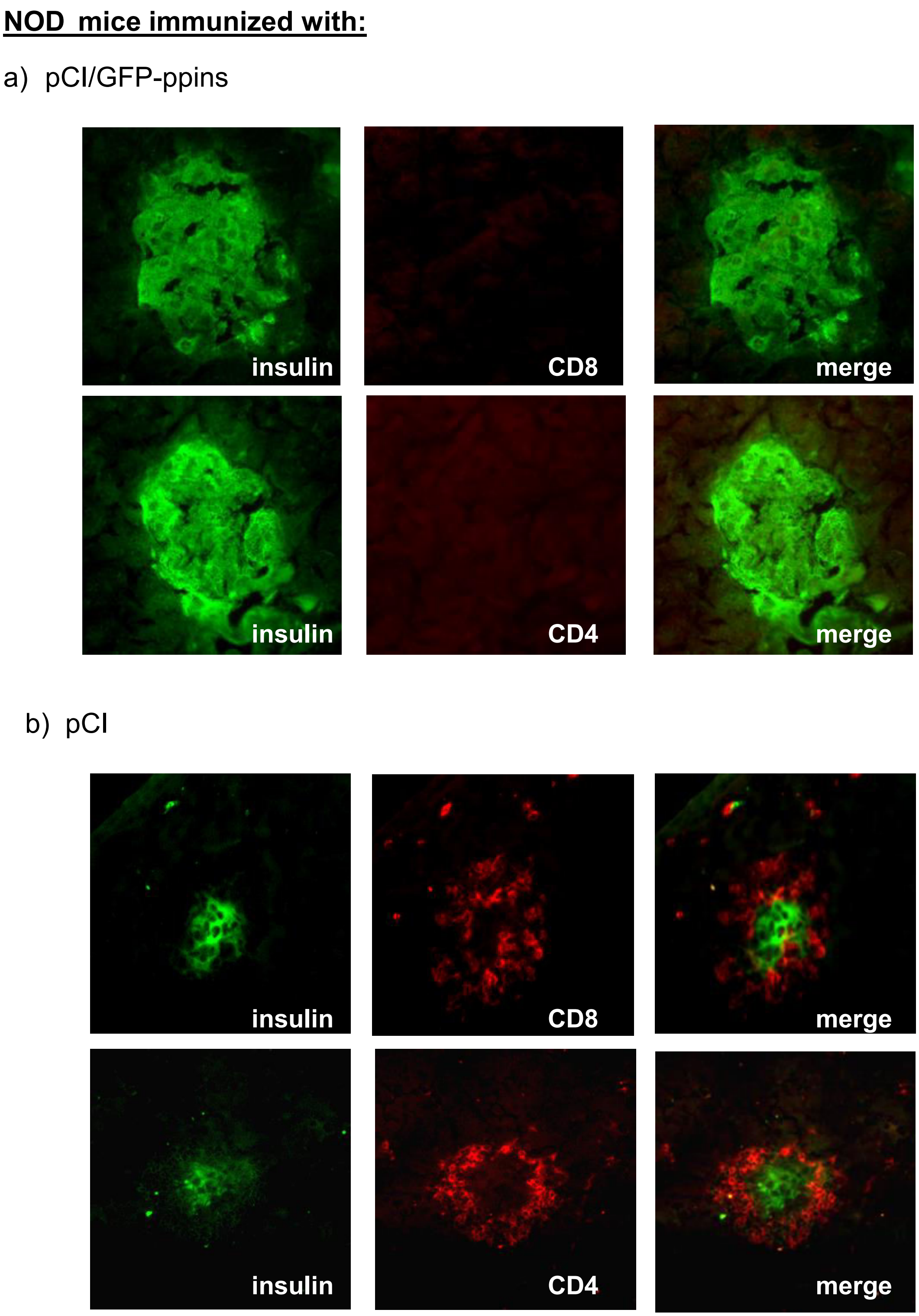

Supplementary Fig. S5

Islet-pathology of pCI/GFP-ppins- and pCI-sham-immunized NOD mice. The islet-pathology and immune cell infiltrations into pancreata of 20 weeks old healthy, pCI/GFP-ppins-immune (a) and control (injected with empty pCI vector) hyperglycemic NOD mice (b) were assessed by immunofluorescence staining with mAbs detecting insulin, $CD8^+$ T cells or $CD4^+$ T cells.
